# Systematic review of model-based cervical screening evaluations

**DOI:** 10.1186/s12885-015-1332-8

**Published:** 2015-05-01

**Authors:** Diana Mendes, Iren Bains, Tazio Vanni, Mark Jit

**Affiliations:** 1 Department of Infectious Disease Epidemiology, Faculty of Epidemiology and Population Health, London School of Hygiene & Tropical Medicine, Keppel Street, Bloomsbury, London, WC1E 7HT UK; 2 Modelling and Economics Unit, Public Health England, 61 Colindale Avenue, London, NW9 5EQ UK; 3 Brazilian Ministry of Health, Esplanada dos Ministérios Bloco G, Brasília-DF, CEP: 70058-900 Brasil

**Keywords:** Systematic review, Human papillomavirus, Cervical cancer, Screening, Mathematical models, Economic evaluations

## Abstract

**Background:**

Optimising population-based cervical screening policies is becoming more complex due to the expanding range of screening technologies available and the interplay with vaccine-induced changes in epidemiology. Mathematical models are increasingly being applied to assess the impact of cervical cancer screening strategies.

**Methods:**

We systematically reviewed MEDLINE®, Embase, Web of Science®, EconLit, Health Economic Evaluation Database, and The Cochrane Library databases in order to identify the mathematical models of human papillomavirus (HPV) infection and cervical cancer progression used to assess the effectiveness and/or cost-effectiveness of cervical cancer screening strategies. Key model features and conclusions relevant to decision-making were extracted.

**Results:**

We found 153 articles meeting our eligibility criteria published up to May 2013. Most studies (72/153) evaluated the introduction of a new screening technology, with particular focus on the comparison of HPV DNA testing and cytology (n = 58). Twenty-eight in forty of these analyses supported HPV DNA primary screening implementation. A few studies analysed more recent technologies - rapid HPV DNA testing (n = 3), HPV DNA self-sampling (n = 4), and genotyping (n = 1) - and were also supportive of their introduction. However, no study was found on emerging molecular markers and their potential utility in future screening programmes. Most evaluations (113/153) were based on models simulating aggregate groups of women at risk of cervical cancer over time without accounting for HPV infection transmission. Calibration to country-specific outcome data is becoming more common, but has not yet become standard practice.

**Conclusions:**

Models of cervical screening are increasingly used, and allow extrapolation of trial data to project the population-level health and economic impact of different screening policy. However, post-vaccination analyses have rarely incorporated transmission dynamics. Model calibration to country-specific data is increasingly common in recent studies.

**Electronic supplementary material:**

The online version of this article (doi:10.1186/s12885-015-1332-8) contains supplementary material, which is available to authorized users.

## Background

Cytological screening for cervical cancer is recognized as having substantially reduced cervical cancer incidence and mortality in many high-income countries (HIC). However, recent technological developments are prompting a paradigm shift in cervical cancer prevention [1]. Human papillomavirus (HPV) DNA testing has greater sensitivity for high-grade lesions than cytology when used as a primary screening method, [2] while a panoply of other biomarkers, such as p16, Ki-67, mRNA, and methylation markers, have been investigated for their potential role in primary screening, triage of borderline cytological outcomes, and triage of HPV-positive results that could enable a fully molecular-based approach to screening [3]. Moreover, where introduced, HPV vaccination is expected to eventually reduce the incidence of cervical cancer and therefore reduce the absolute impact of existing screening programmes, necessitating their reassessment for future unvaccinated and vaccinated cohorts [4].

Hence the choice of optimum cervical screening strategies in future will be highly complex due to the number of technological choices available, combined with epidemiological changes in the target population. Mathematical models offer a way to combine different types of evidence about the choices available (together with their associated uncertainty) to predict the impact of alternative prevention strategies unlikely to be tested in clinical trials due to the enormous time and resource requirements [5]. However, the type of analysis used, the health technologies assessed, and the modelling methods applied may have an important impact on decision-making.

This is the first systematic review encompassing all model-based effectiveness and/or cost-effectiveness analyses of cervical cancer screening strategies. Initial reviews in this area [6,7] only examined cervical cancer models analysing exclusively cytology-based strategies, while those published after 2005 [5,8-10] focused only on economic (and not epidemiological) models. There have been three reviews of HPV DNA testing and cytology for primary screening, [8-10] but only two [9,10] were systematic. Other reviews have also examined HPV DNA testing as triage for equivocal cytological outcomes in high-income settings and visual inspection in low-resource countries, [5] as well as a range of technologies in the USA and in low-resource settings [11]. The limited geographical scope of these reviews and the recent technological development justify a systematic review of the literature, including epidemiological evaluations, over the full range of technologies available in any kind of setting.

The aims of this review are to (i) provide an overview of results from all model-based economic evaluations of cervical screening, in order to inform comprehensive policy making on secondary prevention of cervical cancer, and (ii) identify trends and gaps in these models in order to inform future work.

## Methods

### Search strategy

This review was conducted following guidance of the Centre for Reviews and Dissemination for systematic reviews [12] and Preferred Reporting Items for Systematic Reviews and Meta-Analyses (PRISMA) [13]. We searched the following electronic databases for studies published up to May 2013: MEDLINE®; Embase; Web of Science®; EconLit; the Health Economic Evaluations Database; and The Cochrane Library including the NHS Economic Evaluation Database and the Health Technology Assessment database using the searches strategies in Additional file 1.

### Selection criteria

We included original research articles that met the following criteria:

1. Based on mathematical modelling of HPV infection and/or cervical disease progression
2. Estimated the impact of at least one cervical screening technology/strategy
3. Estimated either clinical outcomes alone (epidemiological models) or both clinical and economic outcomes (economic models)

Studies modelling women of any age at risk of infection, infected, or who had been previously infected with HPV were included, as well as studies on women with concomitant infections (e.g. human immunodeficiency virus (HIV)) or who had been treated for cervical lesions. We included models of HPV vaccination where different cervical screening strategies are compared to each other. Economic evaluations (specifically cost-effectiveness analyses, cost-utility analysis, and cost-benefit analyses) were included if they reported both costs and benefits expected for each strategy of the analysis. Full texts for abstracts and conference presentations identified as potentially relevant in searches were sought, including initiating contact with the corresponding authors when details were otherwise unobtainable. Research articles published in any language in peer-reviewed journals; and abstracts or conference presentations from 2012 onwards published with sufficient details to allow full completion of the pre-established data extraction form were included.

Studies only comparing the costs of different strategies were excluded, as well as publications that were neither (i) archived by the British Library [14] nor (ii) published in a journal included in the Thompson Reuters Impact Factor list [15].

### Study selection

Study selection was performed independently by two reviewers (DM and IB). Initially, the titles and abstracts of the references retrieved in the searches were screened according to the inclusion criteria defined above to identify potentially relevant studies. All titles and abstracts were screened by at least one reviewer; 20% were independently screened by both reviewers. Where initial assessments differed, reviewers’ decisions and disagreements were compared and discussed. Full papers of references identified as potentially relevant in the initial screening were then assessed for eligibility (ten per cent independently assessed by both reviewers). The reviewers again compared results and discussed any differences. A third reviewer (MJ) was consulted where consensus was not reached in any of the screening stages.

### Data extraction

Data extracted included name of first author, year of publication, country of study, type of analysis, type of model, calibration method, strategies/technologies assessed, and main findings. Additional file 2 provides a list of the data extracted. The included studies were grouped by World Health Organization (WHO) region [16] and level of income of the analysed countries, as per the World Bank 2014 income levels [17]. Studies referring to their region of interest as ‘developing countries’ were assumed to relate to all WHO regions.

## Results

**Figure 1:**
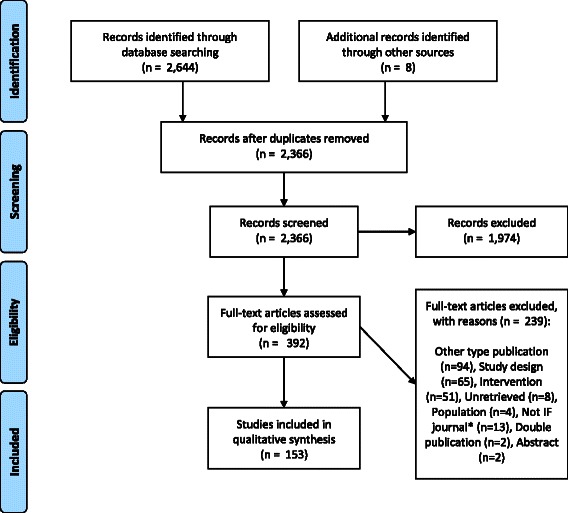
PRISMA Flow diagram of study selection process. *Articles published in journals not included in the British Library catalogue or Thompson Reuters Impact Factor (IF) list.

The searches conducted identified 2,644 studies that potentially met the inclusion criteria set out above. A PRISMA [13] flow diagram of the selection of the included studies is given below (Figure 1) and a completed PRISMA checklist is provided as Additional file 3. From screening titles and abstracts, 392 records were retrieved for full screening, and 153 articles met the inclusion criteria.

Seventy-eight of the 153 publications included in this review explicitly acknowledged that they were adaptations or alternative applications (i.e. without changes to the model assumptions) of previously published models.

**Figure 2:**
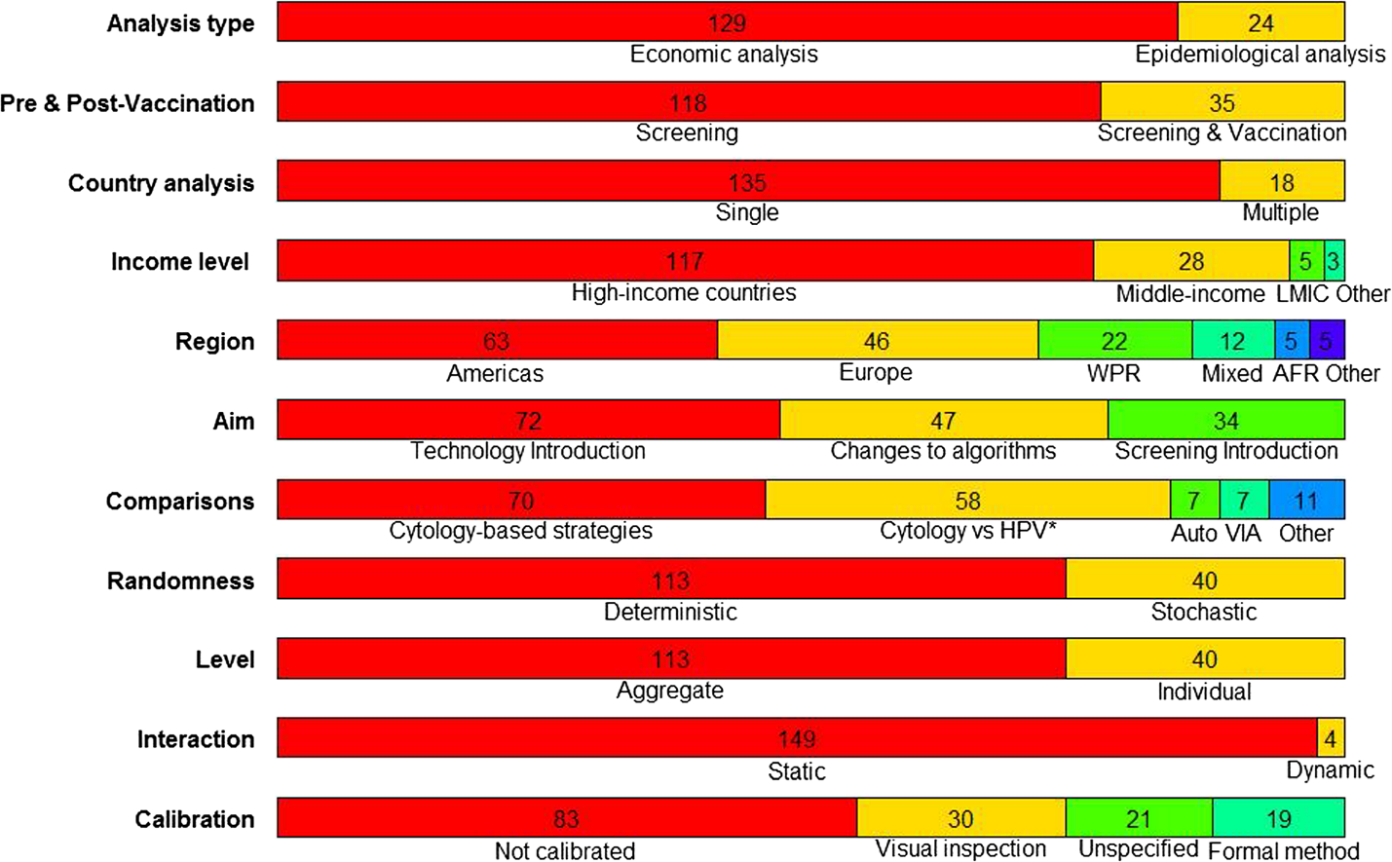
Characteristics of included studies. *Exclusively these technologies; AFR, African Region; Auto; automated cytology; HPV, HPV DNA testing; LMIC, low and middle income countries; VIA, VIA vs HPV DNA testing and cytology; WPR, Western Pacific Region.

The main characteristics of the studies included are summarised in Figure 2 and are discussed further below. Greater detail is provided in Additional files 4 and 5 that present the characteristics of studies that focused on screening alone and on combined screening and vaccination interventions, respectively, by year of publication.

### Countries

**Figure 3:**
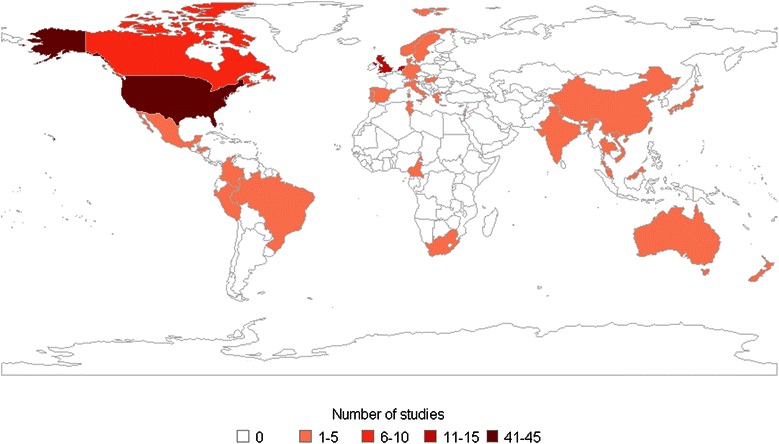
Number of single-country studies per country.

Most included studies (n = 135) were based on a single country. Additional file 6 shows the number of single- and multiple-country studies by country. Forty-five countries were addressed individually (either in single- or multiple-country publications), ten of which – Argentina, Barbados, Belgium, Chile, France, Finland, France, Iceland, Ireland, Kenya, Mozambique, Tanzania, Uganda, and Zimbabwe - were only analysed as part of multiple-country studies. Figure 3 shows the distribution of the included single-country studies on the world map. Over half (80/153) of the studies focused on either the USA (n = 44), the UK (n = 14), the Netherlands (n = 13), or Canada (n = 9). The Americas, Europe, and/or Western Pacific regions accounted for 86% of the studies.

Most studies focused exclusively on HIC (n = 117), whereas 35 studies analysed low- and/or middle-income countries, with 28 analysing only middle-income settings and only 2 studies focusing entirely on low-income ones [18,19]. One study analysed 6 regions of different income-level [20].

### Type of analysis

Most studies (n = 129) included a cost-effectiveness analysis. Of these, 10 presented health outcomes in terms of disease-specific measures only, 79 in terms of lives saved or life years gained, and 40 in terms of the generic health utility measure quality-adjusted life years (QALYs). Quality-adjusted life years were particularly common among studies assessing vaccination alongside screening compared to those which assessed screening alone (42% compared to 21%). There were no cost-benefit analyses (i.e. studies in which both costs and outcomes were expressed in monetary terms).

**Figure 4:**
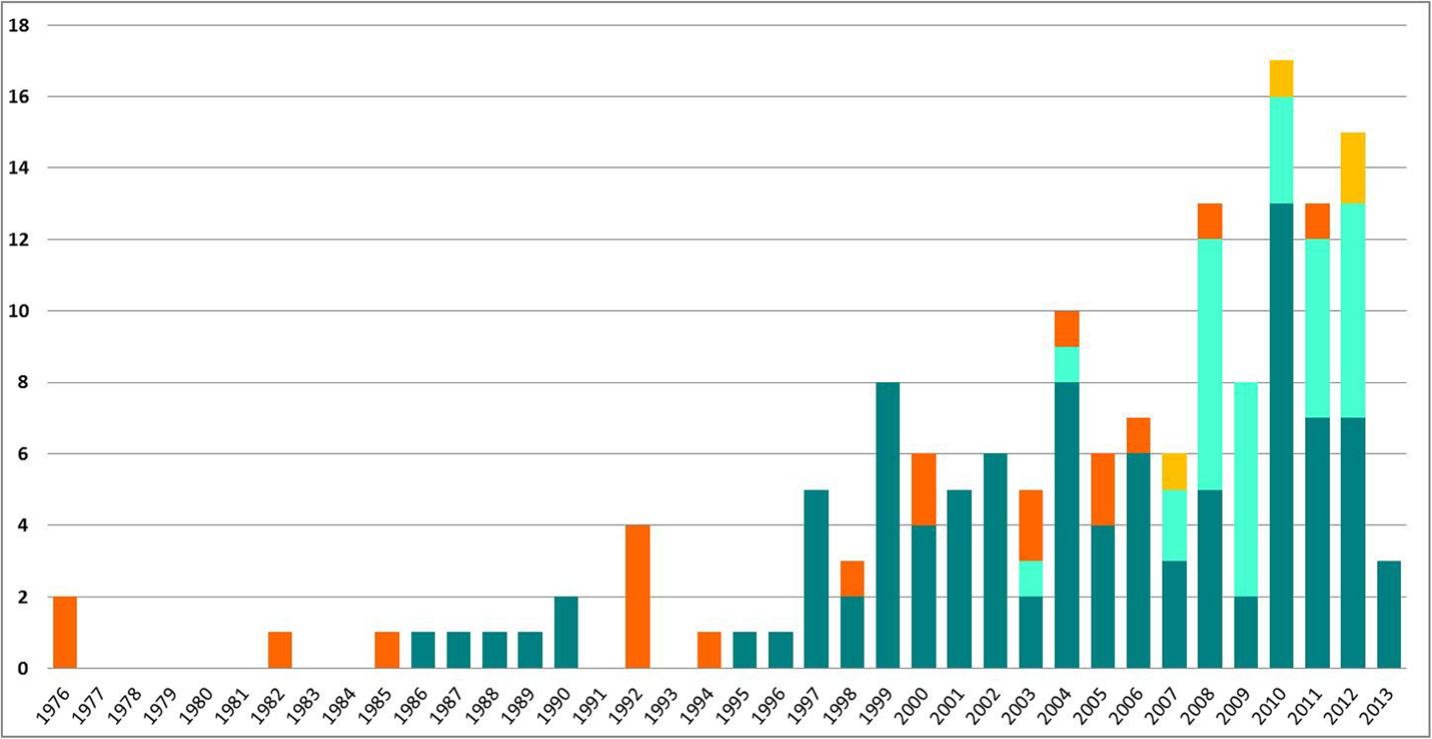
Number of studies by analysis and prevention type over time. Dark blue, Economic Screening; Light blue, Economic Screening & Vaccination; Orange, Epidemiological Screening; Yellow, Epidemiological Screening & Vaccination.

Figure 4 shows the distribution of studies by year according to the type of analysis outcome (epidemiological or economic) and the type of prevention strategies assessed (screening alone or screening combined with vaccination). Post-vaccination economic analyses have become more common in the last decade and economic analyses in general have become dominant compared with studies analysing health outcomes only.

### Type of intervention

The included studies estimated the incremental effectiveness or cost-effectiveness of three types of interventions: (a) introduction of a new screening programme where none existed before (n = 34), (b) changes to existing screening algorithms without the introduction of a new technology (n = 47), and (c) introduction of a new screening technology (n = 72).

- Studies on the impact of introducing a new screening programme (n = 34) were mostly economic evaluations (n = 30). Most were set in middle-income (n = 14) or high-income (n = 13) countries. Several (n = 12) investigated screening strategies post-HPV vaccine introduction. All 34 studies recommended introducing screening.
- Studies exclusively analysing changes to existing screening programmes examined alternative cytology-based strategies (n = 47, 42 in HIC). Most (18/23) studies making recommendations on screening intervals or frequency endorsed an interval of 3 years or more. Recommended starting ages ranged between 20–35 years old, while recommended stopping ages ranged between 60–73 years old. Three studies looked at rescreening cytology negative outcomes, and had mixed results. One study examined follow-up of women post-hysterectomy and recommended against screening women over 40 years [21].
- Seventy-two studies analysed the introduction of a new screening technology to an existing programme. All compared the new technology to cytology apart from one study that compared visual inspection with acetic acid (VIA) to HPV DNA testing. The findings of these comparisons are detailed in the following subsections.

### Technologies assessed

Publications focused on cytology (n = 150), HPV DNA (n = 77), and VIA (n = 12). Overall, the studies analysed 8 screening techniques: cytology (n = 150, of which 34 referred to liquid-based cytology (LBC)), cytology automated reading (e.g. Papnet© and AutoPap©, n = 7), speculoscopy as adjunct to cytology (n = 1), HPV DNA (n = 76), self-sampled HPV DNA testing (n = 4), HPV 16/18 genotyping (n = 1), and VIA (n = 12).

The main technological comparisons made were between (a) alternative cytology-based strategies (n = 77), (b) HPV DNA *versus* cytology (n = 69), (c) VIA *versus* cytology and/or HPV DNA (n = 11). Additional file 7 summarises the findings on comparisons of technologies.

#### Alternative cytology-based strategies

Liquid-based cytology was recommended in 18/26 economic analyses and in one epidemiological analysis comparing it with conventional cytology. The remaining studies recommended conventional cytology (8/27) or were equivocal (1/27).

Automated reading of cytological results was found to be cost-effective when compared to manual reading in all (n = 6) economic studies. One epidemiological study on adding automated reading to LBC concluded that evidence was still insufficient to recommend it relative to manual reading [22]. One economic analysis found the addition of speculoscopy to biennial conventional cytology cost saving and health improving compared with annual conventional cytology alone [23].

#### HPV DNA testing *versus* cytology alone

Several studies examined replacing cytology with HPV DNA testing as the primary screening technique (n = 17) and 15/17 studies found HPV DNA more cost-effective. Twenty-four studies compared co-testing with cytology and HPV DNA (n = 17), or with cytology primary screening only (n = 7). Co-testing was supported in 6/7 studies comparing it with cytology; however, HPV DNA testing was the most supported technology among studies comparing it with co-testing and cytology (10/17), whilst 8/17 were favourable to co-testing, and 6/17 to cytology alone for primary screening (some studies supported more than one technology). Overall, HPV DNA primary screening was supported in 26/34 studies comparing it to cytology alone and/or co-testing.

The introduction of HPV DNA testing to triage minor cytological abnormalities was supported in 9/10 studies comparing it with repeat cytology and immediate referral to colposcopy (7/8), immediate treatment (1/1), or co-testing (1/1) in high- and middle-income countries.

Rapid and relatively-inexpensive HPV DNA testing (careHPV™, n = 3) was found cost-effective in China compared with VIA [24] or cytology, [25] as well as when performed twice a lifetime alongside vaccination compared with once a lifetime without vaccination, provided affordable vaccination cost [26].

Most (3/4) economic analyses of post-treatment screening [21,27-29] investigated the introduction of HPV DNA testing. Two of these recommended its introduction, [27,28] whereas one study found conventional cytology the most cost-effective approach compared to HPV DNA testing or LBC [29].

The introduction of self-sampled HPV DNA primary screening instead of clinic-based HPV DNA testing or conventional cytology was found cost-effective in 2/4 studies that looked at it.

One study on HPV 16/18 genotyping found it cost-effective in the USA for triage of equivocal results of co-testing (HPV DNA and LBC) compared with co-testing alone, HPV DNA with LBC triage, LBC with HPV DNA triage, or LBC alone [30].

#### VIA *versus* HPV DNA and/or cytology

All studies comparing VIA with HPV DNA and/or cytology for primary screening (n = 11) were economic analyses and most comparing HPV DNA testing and VIA (6/9) recommended HPV DNA testing (n = 2) [19,31] or either (n = 4) [18,20,32,33].

One study compared VIA with HPV DNA, cytology, and self-sampling in South Africa and concluded that 1-visit HPV DNA testing was the most effective strategy, slightly more costly than 1-visit VIA [32].

One study only comparing VIA and HPV DNA testing found the latter cost-effective in low resource settings, [19] and all studies comparing VIA with cytology only (n = 2) supported VIA in MIC, [33,34] with one finding cytology cost-effective to screen women over 50 years old every 5 years in Thailand [35].

### Screening and vaccination

Studies analysing screening strategies in vaccinated populations (n = 35) assessed (a) the introduction of screening strategies where non-existent (n = 12), (b) changes to existing cytology-based screening strategies (n = 12), and (c) the introduction of new screening technologies in existing programmes (n = 11).

- Introducing screening (using any technology) alongside vaccination was preferred over screening alone by 10/12 studies (8 regarding low- and/or middle-resource settings).
- Most studies analysing changes to existing cytology-based screening alongside vaccination (10/12, 10 in high- and 2 in middle-income countries) recommended combined screening and vaccination interventions. Half of these studies highlighted the importance of high coverage of screening and immunization programmes. Recommendations on cytology screening target age and interval varied among HIC studies (n = 4).
- Studies on the introduction of screening technologies post-vaccination looked largely at HPV DNA testing and cytology (9/11, 2 in low and middle income countries (LMIC)). HPV DNA testing alone was found more cost-effective than cytology in 5/5 studies focused on primary screening with these technologies alone. Studies comparing these with co-testing as well (n = 3) concluded favourably regarding co-testing [36-38]. One study explored only the introduction of HPV DNA in triage of cytological results, and supported it in the Netherlands, Taiwan, and USA, but not in Canada or the UK [39].

Table 1 summarises the findings and recommendations of the studies included in this review.

**Table 1:** **Summary of findings and recommendations**

Type of intervention	
-	Screening should be introduced (34/34, 100%)
-	Cytology-based screening should have screening intervals ≥3 years (18/23, 78%), starting age ≥25 years old (9/10, 90%), and stopping age ≥60 years old (5/5, 100%)
	- No post-hysterectomy screening follow-up should be given to women >40 years old (1/1, 100%)
**Technologies assessed**	
-	Liquid-based cytology is recommended over conventional cytology (18/27, 67%)
-	Automated reading should be introduced (6/7, 86%)
-	HPV DNA testing for primary screening is more cost-effective than cytology (15/17, 88%)
-	Co-testing is more cost-effective than cytology in HIC (6/7, 86%)
-	HPV DNA testing is supported over co-testing and cytology alone (10/17, 59%)
-	HPV DNA to triage minor cytological abnormalities is endorsed over (i)repeat cytology and immediate colposcopy (7/8), (ii)immediate treatment (1/1), or (iii)co-testing (1/1) (9/10, 90%)
-	HPV DNA testing for post-treatment screening should be introduced (2/3, 67%)
-	Rapid HPV DNA testing should be introduced in China (3/3, 100%)
-	Self-sampled HPV DNA testing as primary screening in HIC is cost-effective *versus* clinic-based HPV DNA or conventional cytology alone(2/2, 100%); however, in upper-middle income countries, it is not cost-effective *versus* other technologies, such as clinic-based HPV DNA (2/2, 100%)
-	HPV 16/18 genotyping should be introduced for triage of equivocal results of co-testing *versus* co-testing alone, HPV DNA with LBC triage, LBC with HPV DNA triage, or LBC alone (1/1, 100%)
-	HPV DNA is more cost-effective than VIA in LMIC (1/1; 100%)
-	VIA is more cost-effective than cytology in LMIC (2/2; 100%)
**Screening and Vaccination**	
-	Screening should be introduced even in a post-vaccination setting (10/12, 83%)
-	Screening should be continued after vaccination is introduced (10/12, 83%)
-	Post-vaccination HPV DNA primary screening is cost-effective compared to cytology alone in HIC (5/5, 100%)

Figures in parentheses show the proportion (x/y) and percentage (%) of relevant studies supporting each recommendation.

### Modelling methods

The modelling approaches used in the included studies were classified according to the following dimensions [40]:

- Randomness (stochastic *versus* deterministic)

In deterministic models, events such as HPV acquisition and clearance occur at a pre-determined rate. Stochastic models incorporate randomness (stochasticity) in the occurrence of these events, so the outcomes of a model are not exactly the same each time it is run.

(b) Level (individual *versus* aggregate)

Individual-based models simulate and record the events that occur in each modelled individual’s lifetime, so that each individual has unique characteristics. In contrast, aggregate models group individuals with similar characteristics into compartments, eliminating their variability within each compartment. Hence individual-based models capture population heterogeneity more easily.

(c) Interaction (static *versus* dynamic)

If the rate at which people get infected with HPV (i.e. the force of infection) is likely to change, such as following population-based vaccination, then herd immunity (i.e. indirect protection of susceptible individuals by a significant proportion of immune individuals in the population) is likely to affect the model results greatly. Dynamic models account for herd immunity as the risk of infection is modelled as dependent on the number of infectious individuals rather than assumed to be constant over time (static models).

The models found were mainly static (149/153), deterministic (113/153) and aggregate (113/153); all aggregate models were deterministic. Only 4 studies were dynamic and all of these were deterministic and modelled individuals at an aggregate level. Three of the four dynamic models found were used to assess screening strategies alongside vaccination. Similarly to models of screening interventions alone, the models used for post-vaccination analyses were mainly static (32/35), and deterministic aggregate (19/35). Stochastic individual-based models were more common among post-vaccination analyses (16/35; 46%) than amid those analysing screening interventions alone (24/118; 20%).

Many models require values of parameters that are difficult to measure directly, such as the rate of progression from CIN3 to invasive cancer. Such values can be estimated by calibrating the model, that is, adjusting its internal parameters until model outputs (such as cancer incidence) match observational data. The extent to which the outputs can match data is often quantified using a goodness-of-fit measure. Commonly used quantitative goodness-of-fit measures include the sum of squared residuals, the chi-squared statistic and the likelihood of the data [41].

Most studies (n = 83) did not report having calibrated their models at all. Of those that reported calibration (n = 70), 21 did not specify the goodness-of-fit measure used and 30 only assessed model fit to data visually without using any quantitative goodness-of-fit measure. The remaining studies (n = 19) explicitly reported using a formal goodness-of-fit measure. A greater proportion of models used for the assessment of screening strategies alongside vaccination were calibrated (23/35; 66%) compared with those of models only assessing screening strategies (47/117; 40%).

## Discussion

Many studies addressing a wide range of questions met our inclusion criteria compared to that in other cervical cancer-related reviews [42,43]. This may reflect the substantial global burden of cervical cancer, the recent development of new screening methods and technologies, as well as the role mathematical modelling has played regarding context-specific policy questions that only very large long term trials would address [43,44].

### Results from model-based evaluations of cervical screening

Most studies included a cost-effectiveness analysis (129/153) and investigated the introduction of new screening technologies (72/153), with fewer focusing exclusively in alternative strategies using already-adopted technologies (47/153), and even fewer on the introduction of screening programmes where non-existent (34/153). Evaluations of the introduction of a screening technology were generally favourable to its adoption, with LBC recommended *over* conventional cytology (18/27), HPV DNA recommended *over* cytology for primary screening (15/17), rapid HPV DNA (3/3) or self-sampling (2/4) recommended for primary screening, and HPV DNA (9/10) or genotyping (1/1) recommended for triage of equivocal results.

Overall, our findings are in line with those of previous reviews of cost-effectiveness analyses [5,8-11] and post-vaccination analyses in the context of developed countries with existing screening programs [40], which mostly recommend the introduction of HPV DNA primary screening in high-resource settings and the revision of screening policies towards the introduction of HPV DNA primary testing.

As Nahvijou and colleagues also found, [10] there is a discrepancy between guidelines and model-based evaluations regarding more recent technologies. Generally, current HIC screening guidelines ( Summary of cervical screening guidelines provided in Additional file 8) are aligned with the overall findings of evaluations of cytology-based strategies; however, most concluded lacking sufficient evidence on the effectiveness of HPV DNA testing for primary screening to support its implementation, [45] with only a few countries, such as Australia, the Netherlands and the USA, recommending it at the moment.

### Trends and gaps identified

Most of the global cervical cancer burden lies in low- and middle-income countries without organised screening programmes [46] However, as noted in previous reviews, [5,11] only a small proportion of studies in our review (34/153) addressed these settings, with the vast majority (33/34) supporting the existence of a screening programme. Indeed, over half the studies (80/153) were set in just 4 HIC – the USA, the UK, the Netherlands, or Canada. More evaluations focused on the regions with the greatest cervical cancer burden may have greater influence in driving adoption of screening technologies where they are most needed.

Currently several molecular biomarkers are being investigated for their potential to be integrated alongside cytology and HPV DNA testing in screening algorithms. However, no model-based study was found in this review on these emerging screening technologies. Only a few studies analysed more recent technologies as rapid HPV DNA testing, self-sampled HPV DNA testing, or HPV 16/18 DNA genotyping. No study on rapid HPV DNA testing was found in a low-income setting either.

Some molecular-based tests are thought to have the potential to improve cytology’s accuracy and reproducibility (e.g. p16 immunostaining), while other are thought to be promising alternatives to cytology (e.g. HPV DNA testing, HPV mRNA testing, p16/ki-67 dual immunostaining, or methylation markers) as they can be subject to automated quantification [47]. The clinical utility of HPV DNA testing has been shown, [2] and it has recently been introduced in primary screening in a few HIC, e.g. the Netherlands and Ontario [48]. These recent developments in screening technologies may suggest a transition to a fully molecular-based screening approach. However, the population-level effectiveness and cost-effectiveness behind many of the molecular technologies is still unexplored. For most biomarkers there is currently only cross-sectional evidence of their potential accuracy [3]. HPV mRNA testing for instance has been recently approved by the U.S. Food and Drug Administration for screening women over 30 years in combination with cytology, despite evidence from longitudinal trials of its improved accuracy in the detection of CIN2+ lesions who do not regress be not yet available [49]. Mathematical models are a key tool to allow results from trials and observational studies of these technologies to be extrapolated to explore their long-term impact in population-based screening programmes.

Another aspect of research that can be explored via mathematical modelling is the interaction between vaccination and screening. Vaccinating adolescent girls has been found likely to be cost-effective even in settings with existing screening programmes [40,50]. However, vaccination is expected to decrease the incidence of cervical abnormalities and eventually cancer [51]. Hence the positive predictive value of cytology will decrease, as will the effectiveness of most screening modalities. [43,52] In order to assist in population level policy making, future analyses in settings with vaccination will need to account for its impact on existing and prospect screening programmes. This is particularly true if a 9-valent HPV vaccine is successful in trials, as it is projected to ultimately prevent 90% of invasive cervical cancers [53].

Also, most models of screening in post-vaccination settings relied on a static infection structure. This may be suitable for comparing alternate screening strategies in a setting in which disease prevalence is constant, but would not capture the long-term changes in HPV prevalence, in settings with successful national HPV vaccination programmes [54] such as the UK, Australia and Portugal. Dynamic transmission models are particularly important now that a 9-valent HPV vaccine has shown high immunogenicity and efficacy in clinical trials [55]. This will have further implications on cervical screening since vaccinated girls will have a very low risk of infection with an oncogenic HPV type and hence risk of cervical cancer. The few dynamic models compared alternative cytology-based strategies [56,57] or strategies with rapid HPV DNA testing *versus* vaccination only or alongside vaccination [26]. Their overall results were consistent with those of static models in that screening strategies alongside vaccination maximise health outcomes. However, it can take many years for the direct and indirect impact of vaccination to be observed in surveillance data, so dynamic models will be increasingly important to explore changes to screening as the first vaccinated cohorts enter the age of screening eligibility.

Model calibration to observed setting-specific data has become more common; however it is still not routinely used. As most natural history parameters governing the progress of cervical abnormalities are very difficult to measure directly, model calibration enables their estimation based on observable outcomes such as abnormal screening results. This is generally a more reliable approach than making assumptions on parameters based on limited studies, often in unrepresentative populations [41,58]. Even the studies reporting having calibrated these parameters to outcome data often gave few details about the goodness-of-fit measure used and very rarely provided details on other aspects of calibration, such as the selection of calibration targets, parameter search strategies, and convergence criteria used. Detailed reporting of the calibration process should be common practice for reproducibility purposes [59]. Also, there should be an indication of uncertainty in the parameter estimates used and how it is incorporated to judge the sensitivity of model predictions to the data sources used.

This review is subjected to limitations. We focused on models used to assess the impact of alternative screening strategies, and excluded model-based studies assessing vaccination strategies, including those modelling screening strategies alongside vaccination that did not compare different screening strategies. Because of the volume and diversity of the relevant modelling literature, we did not critically appraise the quality of individual studies, but instead focused on providing an overview of the main approaches and conclusions of the models. Further work is needed to critically review modelling literature that addresses specific questions (such as the choice between cytological and DNA-based screening methods) in more detail. The main strength of our work lies in providing a broad overview of the vast literature over a long time period, and in identifying key conclusions that are common across models as well as gaps in the methodology and scope of current models.

## Conclusions

The main questions addressed over time by models used to assess cervical cancer screening strategies focused on high-income settings analysing matters relevant to LMIC as well, such as the introduction of HPV DNA testing and more recently the most appropriate post-vaccination screening strategy. Despite the increasingly large number of publications, few studies investigated the utility of HPV DNA self-sampling and genotyping in future screening programmes, and none explored the potential role of emergent molecular markers. Transmission dynamics have rarely been incorporated and model calibration is not standard practice yet. Dynamic models fitted to country-specific data could be helpful tools to investigate future post-vaccination screening strategies.

## Additional files

Additional file 1:
**Search strategies for each database consulted.**

Additional file 2:
**List of the types of data extracted and categorisation used.**

Additional file 3:
**Completed PRISMA 27-item checklist for current systematic review.**

Additional file 4: **Main characteristics of studies focused on screening interventions alone.** Table with main data extracted for included studies on screening interventions alone.
Additional file 5: **Main characteristics of studies focused on screening interventions alongside vaccination.** Table with main data extracted for included studies on screening and vaccination interventions.
Additional file 6: **Number of single- and multiple-country studies per country.** Table with number of studies by country and study type.
Additional file 7:
**Summary of findings on comparisons of screening technologies.**

Additional file 8:
**Summary of cervical screening guidelines.**

## Abbreviations

DNA: Deoxyribonucleic acid
HIC: High-income countries
HIV: Human immunodeficiency virus
HPV: Human papillomavirus
LBC: Liquid-based cytology
LMIC: Low- and middle-income countries
mRNA: Messenger ribonucleic acid
PRISMA: Preferred Reporting Items for Systematic Reviews and Meta-Analyses
QALY: Quality-adjusted life year
UK: United Kingdom
USA: United States of America
VIA: Visual inspection with acetic acid
WHO: World Health Organization
