# Supplementary material for: Systematic review of model-based cervical screening evaluations
Source: BMC Cancer. 2015 May 1;15:334. doi: 10.1186/s12885-015-1332-8 (PMC4419493; doi:10.1186/s12885-015-1332-8)
Supplement: Additional file 1: — Search strategies for each database consulted. [file 12885_2015_1332_MOESM1_ESM.docx]

**Additional material1. Searches strategies**

| Database and search date | Search strategy |
| --- | --- |
| Medline  1946 to May Week 5 2013 (OvidSP)  11/06/2013 (updated - extended headings) | **1. exp Uterine Cervical Dysplasia/ or exp Uterine Cervical Diseases/ or exp Cervical Intraepithelial Neoplasia/ or exp Uterine Cervical Neoplasms/**  **2. (cervix or cervical or cervico*) (tw)**  **3. cancer* (tw) or carcinoma.mp. or adenocarcinoma.mp. or neoplas* (tw) or dysplas* (tw) or dyskaryos* (tw) or squamous (tw) or CIN (tw) or CINII* (tw) or CIN2* (tw) or CINIII* (tw) or CIN3* (tw) or SIL (tw) or HSIL (tw) or H-SIL (tw) or LSIL (tw) or L-SIL (tw) or ASCUS (tw) or AS-CUS (tw) [mp=title, abstract, original title, name of substance word, subject heading word, keyword heading word, protocol supplementary concept, rare disease supplementary concept, unique identifier]**  **4. 2 and 3**  **5. 1 or 4**  **6. HPV.mp. or Tumor Virus Infections/ or Papillomavirus Infections/ or Oncogene Proteins, Viral/ or Papillomaviridae/ or human papilloma.mp. or Alphapapillomavirus/**  **7. (cytolog* or liquid based cytology) (tw)**  **8. (hybrid capture or (HC2 or HCII or HC 2 or HC II)).mp.**  **9. (pap or papanicolaou or vagina* or cervical or cervix or cervico*) (tw) or Vaginal smears/**  **10. smear*.mp. or test (tw) or tests (tw) or testing (tw) or tested (tw) or swab*.mp. or scrap*.mp. [mp=title, abstract, original title, name of substance word, subject heading word, keyword heading word, protocol supplementary concept, rare disease supplementary concept, unique identifier]**  **11. 9 and 10**  **12. Early Detection of Cancer/**  **13. DNA, Viral/**  **14. PCR.mp. or Polymerase Chain Reaction/**  **15. colposcopy.mp. or Colposcopy/ or visual inspection.mp.**  **16. (oncogene mRNA or methylation or proliferation or integration).mp. or immunohistochemistry/ or Tumor Markers, Biological/ or Ki-67 Antigen/ or ki67.mp. or Tumor Suppressor Protein p53/ or Antibodies, Monoclonal/ or Cyclin-Dependent Kinase Inhibitor p16/ or p16.mp. or E6.mp. or E7.mp. or 3q.mp. or 5p.mp. or MCM?.mp. or Top2A.mp. or CDC6.mp. or DAPK1.mp. or CADM1.mp. or RARB.mp.**  **17. Enzyme-Linked Immunosorbent Assay/**  **18. antibod*.mp. or Antibod*/**  **19. models, theoretical/ or models, biological/ or exp models, statistical/ or likelihood functions/ or linear models/ or logistic models/ or exp models, economic/ or nomograms/ or proportional hazards models/ or nonlinear dynamics/ or Cost-Benefit Analysis/ or Epidemiologic methods/ or mathematical concepts/ or health care evaluation mechanisms/**  **20. 5 or 6**  **21. 7 or 8 or 11 or 12 or 13 or 14 or 15 or 16 or 17 or 18**  **22. screen*.mp. or Mass Screening/ or Triage/ or management.mp. or follow up.mp. or marker.mp. or biomarker.mp.**  **23. 21 or 22**  **24. 19 and 20 and 23**  **25. limit 24 to humans** |
| Embase Classic+Embase  1947 to 2013 May 9 (OvidSP)  10/05/2013 | **1. papilloma*.mp. or exp Papilloma virus/ or HPV.mp. or Wart virus/ or alphapapillomavirus/**  **2. cervical cancer.mp. or exp uterine cervix carcinoma in situ/ or exp uterine cervix tumor/ or exp uterine cervix cancer/ or exp uterine cervix dysplasia/ or exp uterine cervix hypertrophy/ or exp uterine cervix disease/ or exp uterine cervix carcinoma/ or ((cervic*.mp. or exp cervix/) and (exp squamous cell carcinoma/ or exp cancer/ or exp carcinoma/ or exp neoplasia/ or exp neoplasm/ or exp dysplasia/)) or CIN.mp. or SIL.mp. or ASCUS.mp.**  **3. exp vagina smear/ or exp uterine cervix cytology/ or exp Papanicolaou test/ or cervical cancer screening.mp.**  **4. screen*.mp. or Mass Screening/ or triage.mp. or management.mp. or follow up.mp. or marker.mp. or biomarker.mp.**  **5. DNA/ or PCR.mp. or Polymerase Chain Reaction/ or colposcopy.mp. or Colposcopy/ or Visual inspection.mp. or (oncogene mRNA or methylation or integration or proliferation).mp. or immunohistochemistry/ or Tumor Markers, Biological/ or Ki-67 Antigen/ or ki67.mp. or Tumor Suppressor Protein p53/ or Antibodies, Monoclonal/ or Cyclin-Dependent Kinase Inhibitor p16/ or p16.mp. or E6.mp. or E7.mp. or 3q.mp. or 5p.mp. or MCM?.mp. or Top2A.mp. or Enzyme-Linked Immunosorbent Assay/ or CDC6.mp. or DAPK1.mp. or CADM1.mp. or RARB.mp. or antibod*.mp. or Antibod*/**  **6. computer model/ or statistical model/ or stochastic model/ or loglinear model/ or biological model/ or theoretical model/ or process model/ or hidden Markov model/ or mathematical model/ or proportional hazards model/ or population model/ or compartment model/**  **7. 1 or 2**  **8. 3 or 5**  **9. 4 and 8**  **10. 6 and 7 and 9**  **11. limit 10 to human** |
| Econlit  1961 to April 2013 (OvidSP)  10/05/2013 | **1. cervi*.mp. [mp=heading words, abstract, title, country as subject]**  **2. HPV.mp. [mp=heading words, abstract, title, country as subject]**  **3. papilloma*.mp. [mp=heading words, abstract, title, country as subject]**  **4. (papanicol* or smear or cytolog* or liquid?based cytolog*).mp. [mp=heading words, abstract, title, country as subject]**  **5. (screen* or test* or inspection or colposcop* or DNA or triage or biomarker* or RNA or methilation or integration or proliferation or p16 or antibod*).mp. [mp=heading words, abstract, title, country as subject]**  **6. 1 or 2 or 3**  **7. 4 or 5**  **8. 6 and 7**  **9. model*.mp. [mp=heading words, abstract, title, country as subject]**  **10. 8 and 9** |
| HEED  10/05/2013 | **AX= 'HPV'**  **AX= 'CERVICAL' Or 'CERVICAL-CANCER' Or 'CERVICAL-CANCER-SCREENING' Or 'CERVICAL-VAGINAL' Or 'CERVICAL/VAGINAL' Or 'CERVICO-VAGINAL' Or 'CERVICOVAGINAL'**  **AX= 'SCREEN' Or 'SCREEN-ALL'**  **AX= 'MODEL' Or 'MODEL-BASED' Or 'MODEL-CALCULATED' Or 'MODEL-DERIVED' Or 'MODEL-ESTIMATED' Or 'MODEL-GENERATED' Or 'MODEL-PREDICTED' Or 'MODEL-PROJECTED' Or 'MODEL-SIMULATED' Or 'MODELLING-TECHNIQUES' Or 'MODELLING/DECISION-ANALYTIC' Or 'MODELLING' Or 'MODELS-STATISTICAL'**  **CS= 1 OR 2**  **CS= 3 AND 4 AND 5** |
| Cochrane Library  (all databases apart from CENTRAL, no limits)  10/05/2013 | **ID Search**  **#1 MeSH descriptor: [Uterine Cervical Neoplasms] explode all trees**  **#2 MeSH descriptor: [Papillomaviridae] explode all trees**  **#3 HPV**  **#4 MeSH descriptor: [Mass Screening] explode all trees**  **#5 model***  **#6 {or #1-#3}**  **#7 {and #4-#6}** |
| Web of Science  14/05/2013 | **Topic=(Cervi*) AND Topic=(cancer or carcinoma or neoplas*or dysplas* or tumor or tumour or hypertrophy or disease or squamous or CIN or SIL or ASCUS)**  **OR**  **Topic=(HPV or human papilloma*)**  **AND**  **Topic=(screen* or test*) AND Topic=(smear or pap* or cytology or visual inspection or VIA or marker or DNA or colposcopy or RNA or methylation or integration or proliferation or immunohistochemistry or Ki-67 antigen or p53 or antibod* or p16 or E6 or E7 or 3q or 5p or MCM? or Top2a or CDC6 or DAKP1 or CADM1 or RARB)**  **AND**  **Topic=(model*) AND Topic=(math* or computer or statistic* or likelihood or linear or logistic or economic)** |
