# Supplementary material for: Systematic review of model-based cervical screening evaluations
Source: BMC Cancer. 2015 May 1;15:334. doi: 10.1186/s12885-015-1332-8 (PMC4419493; doi:10.1186/s12885-015-1332-8)
Supplement: Additional file 2: — List of the types of data extracted and categorisation used. [file 12885_2015_1332_MOESM2_ESM.docx]

**Additional material 2. List of data extracted variables**

| **Administrative variables** | | |
| --- | --- | --- |
| RefID | *Reference identification number* | |
| Status | *Retrieved; unretrieved* |  |
| PubType | *Journal article; review; comment* | |
| Reviewer | *Reviewer’s name* |  |
| Decision | *Include; exclude; unclear* |  |
| Reviewer comments |  |  |
| **Variables** |  |  |
| Author | *First author* |  |
| Year |  |  |
| Title |  |  |
| Language |  |  |
| Country |  |  |
| Population | *Gender, age, comorbidities* | |
| HIV | *y/n* |  |
| **Technologies** |  |  |
| Intervention | *Strategies assessed* | |
| Comparator | *Baseline strategy* | |
| **Model type** |  |  |
| **Study Aim** |  |  |
| **Outcomes** |  |  |
| Results | *Main conclusion (1-2 sentences)* | |
| **Calibration** |  |  |
| **Form drop-down lists by category** | | |
| **Technologies** |  | **Study Aim** |
| Unspecified |  | Effectiveness of screening strategies/interventions |
| Cytology |  | Effectiveness of vaccination strategies/vaccines |
| - Conventional cytology | | Effectiveness of treatment strategies/interventions |
| - LBC |  | Cost-effectiveness of screening strategies/interventions |
| - Automated reading |  | Cost-effectiveness of vaccination strategies/vaccines |
| HPV DNA |  | Cost-effectiveness of treatment strategies/interventions |
| Self-sampled HPV DNA |  | Parameter estimation |
| VIA |  | Other |
| VILI |  |  |
| Colposcopy |  | **Outcomes*** |
| Vaccine |  | HPV Prevalence |
|  |  | Number of cervical cases |
| **Model type** |  | Number of deaths avoided |
| Deterministic |  | Cost per cervical case |
| Stochastic |  | Cost per death avoided |
| Aggregate |  | Cost per life year gained |
| Individual-based |  | Cost per QALY gained |
| Static |  | Cost per DALY averted |
| Transmission dynamic |  | Incidence of cervical cases |
| Hybrid |  | Net Benefit |
| Compartmental/state transition/Markov | | Cost and Lys |
| Decision tree |  | Cost per vaccinated girl (CVG) |
|  |  | Number of tests/repeats |
| **Calibration** |  | Life years saved |
| Not reported |  | Smears per LYG |
| Visual inspection |  | DALYs averted |
| Least squares |  | Risk reduction |
| Chi-squared |  |  |
| Likelihood |  |  |
| Other |  |  |
| Unspecified method |  |  |
