# Supplementary material for: Systematic review of model-based cervical screening evaluations
Source: BMC Cancer. 2015 May 1;15:334. doi: 10.1186/s12885-015-1332-8 (PMC4419493; doi:10.1186/s12885-015-1332-8)
Supplement: Additional file 4: — Main characteristics of studies focused on screening interventions alone. Table with main data extracted for included studies on screening interventions alone. [file 12885_2015_1332_MOESM4_ESM.docx]

**Additional material 4. Main characteristics of studies focused on screening interventions alone (n=118)^[[1]](#footnote-1)^**

| First Author | Year | Country | Econ/Epid | Aim | Technologies | Main findings | Type of model | Calibration |
| --- | --- | --- | --- | --- | --- | --- | --- | --- |
| Agorastos[1] | 2010 | Greece | Econ | TechIntro | Cyt vs HPV | HPV/(Cyt&Colp)+ | Static, Deterministic, Aggregate | Not reported |
| Anderson[2] | 2008 | Australia | Econ | Algorithm | Cyt vs Cyt | Current 2y(20-69);25-; 74-;1y-;3y? | Static, Deterministic, Aggregate | Visual inspection |
| Andres-Gamboa[3] | 2008 | Colombia | Econ | TechIntro | Cyt vs HPV | HPV/Pap5y+ | Static, Deterministic, Aggregate | Not reported |
| Atashili[4] | 2011 | Cameroon | Epid | ScreenIntro | Cyt vs Cyt | HAART+Cyt1x+ | Static, Deterministic, Aggregate | Not reported |
| Balasubramanian[5] | 2010 | USA | Econ | TechIntro | Cyt vs HPV vs SS | OrgScreen+;HPV-; SS/LBC3y+ | Static, Deterministic, Aggregate | Not reported |
| Berkhof[6] | 2006 | Netherlands | Econ | TechIntro | Cyt vs HPV | ConvC-, LBC/HPV+ | Static, Deterministic, Aggregate | Not reported |
| Berkhof[7] | 2010 | Netherlands | Econ | TechIntro | Cyt vs HPV | HPV/Pap+ | Static, Stochastic, Individual | Formal method |
| Bidus[8] | 2006 | USA | Econ | TechIntro | Cyt vs HPV | LBC/HPV2y+ | Static, Deterministic, Aggregate | Unspecified method |
| Bistoletti[9] | 2008 | Sweden | Econ | TechIntro | Cyt vs HPV | OrgPap+, Pap/HPV9y+ | Static, Deterministic, Aggregate | Not reported |
| Boyd[10] | 1989 | UK | Epid | Algorithm | Cyt vs Cyt | OrgScreen+, >=3y+ | Static, Stochastic, Individual | Formal method |
| Brown[11] | 1999 | USA | Econ | TechIntro | Cyt vs Auto | ConvC&Auto3y+ | Static, Deterministic, Aggregate | Not reported |
| Burger[12] | 2012 | Norway | Econ | TechIntro | Cyt vs HPV | HPV/LBC >=34+ | Static, Stochastic, Individual | Formal method |
| Campos[13] | 2012 | USA | Econ | TechIntro | Cyt vs HPV | LBC/LBC-, LBC/Colp(<21, >25)+, LBC/HPV(21-24)+ | Static, Deterministic, Aggregate | Not reported |
| Canfell[14] | 2004 | UK | Epid | Algorithm | Cyt vs Cyt | 3y(25-50)5y(50-64)+ | Static, Deterministic, Aggregate | Visual inspection |
| Chow[15] | 2010 | Taiwan | Econ | TechIntro | Cyt vs HPV | HPV/Cyt5y+ | Static, Deterministic, Aggregate | Not reported |
| Chuck[16] | 2010 | Canada | Econ | TechIntro | Cyt vs HPV | Pap/HPV/Pap(>30)+; LBC- | Static, Deterministic, Aggregate | Unspecified method |
| Coppleson[17] | 1976 | USA | Epid | Algorithm | Cyt vs Cyt | Screen(34-73)10x+ | Static, Deterministic, Aggregate | Not reported |
| Coupe[18] | 2007 | Netherlands | Econ | TechIntro | Cyt vs HPV | HPV6mth + HPV&Pap24mth+ | Static, Deterministic, Aggregate | Not reported |
| Creighton[19] | 2010 | Australia | Econ | Algorithm | Cyt vs Cyt | 3y+ | Static, Deterministic, Aggregate | Unspecified method |
| de Bekker-Grob[20] | 2012 | Netherlands | Econ | TechIntro | Cyt vs HPV | LBC>ConvC +/- | Static, Stochastic, Individual | Visual inspection |
| de Kok[21] | 2012 | European Union | Econ | TechIntro | Cyt vs HPV | HPV/Cyt+ | Static, Stochastic, Individual | Not reported |
| Dewilde[22] | 2004 | UK, USA, Australia, Japan | Econ | Algorithm | Cyt vs Cyt | 3y+ | Static, Deterministic, Aggregate | Visual inspection |
| Eddy[23] | 1987 | USA, Canada, Europe | Epid | Algorithm | Cyt vs Cyt | 3y+ | Static, Deterministic, Aggregate | Visual inspection |
| Fennessy[24] | 2002 | Australia | Econ | TechIntro | Cyt vs Cyt | LBC- | Static, Deterministic, Aggregate | Not reported |
| Fetters[25] | 2003 | USA | Econ | Algorithm | Cyt vs Cyt | ScreenPostHyst- | Static, Deterministic, Aggregate | Not reported |
| Flores[26] | 2011 | Mexico | Econ | TechIntro | Cyt vs HPV vs SS | HPV+;HPV&Cyt+; SS- | Static, Deterministic, Aggregate | Unspecified method |
| Frame[27] | 1998 | Europe and North America | Epid | Algorithm | Cyt vs Cyt | IncCov+;>=3y+; IncFreq- | Static, Deterministic, Aggregate | Not reported |
| Goldie[28] | 2004 | USA | Econ | TechIntro | Cyt vs HPV | ConvC-, LBC+, LBC/HPV=HPV&LBC(>30)3y+ | Static, Deterministic, Aggregate | Visual inspection |
| Goldie[29] | 2001 | USA | Econ | TechIntro | Cyt vs HPV | HPV&Cyt+ | Static, Deterministic, Aggregate | Not reported |
| Goldie[30] | 2005 | India, South Africa, Kenya, Peru, Thailand | Econ | ScreenIntro | Cyt vs HPV vs VIA | Pap-, HPV+, VIA+ | Static, Deterministic, Aggregate | Visual inspection |
| Goldie[31] | 2001 | South Africa | Econ | ScreenIntro | Cyt vs HPV vs SS vs VIA | VIA+, 1-visit HPV+, SS+vsNoScreen | Static, Deterministic, Aggregate | Unspecified method |
| Goldie[32] | 1999 | USA | Econ | Algorithm | Cyt vs Cyt | HIV:Cyt1y+ | Static, Deterministic, Aggregate | Unspecified method |
| Gustafsson[33] | 1992 | Sweden, Canada, USA, UK, Barbados | Epid | Algorithm | Cyt vs Cyt | OrgScreen+; (30-60)or (31-67)5x+ | Static, Deterministic, Aggregate | Not reported |
| Gutierrez-Aguado[34] | 2011 | Peru | Econ | ScreenIntro | Cyt vs Cyt | ScreenAlone+ | Static, Deterministic, Aggregate | Not reported |
| Gyrd-Hansen[35] | 1995 | Denmark | Econ | ScreenIntro | Cyt vs Cyt | 4y(25-59)+ | Static, Deterministic, Aggregate | Unspecified method |
| Hadwin[36] | 2008 | UK | Econ | Algorithm | Cyt vs Cyt | Pap/Colp(1mth)+ | Static, Deterministic, Aggregate | Not reported |
| Helfand[37] | 1992 | USA | Epid | Algorithm | Cyt vs Cyt | Improve Pap quality+ (reduce false-negative rate) | Static, Deterministic, Aggregate | Not reported |
| Hughes[38] | 2005 | USA | Econ | TechIntro | Cyt vs HPV | LBC+, LBC/HPV+ | Static, Deterministic, Aggregate | Not reported |
| Hutchinson[39] | 2000 | USA | Econ | TechIntro | Cyt vs Auto | ConvC-, Auto+, LBC+ | Static, Deterministic, Aggregate | Unspecified method |
| Karnon[40] | 2004 | UK | Econ | TechIntro | Cyt vs Cyt | LBC+ | Static, Deterministic, Aggregate | Not reported |
| Kim[41] | 2004 | Hong Kong | Econ | ScreenIntro | Cyt vs Cyt | LBC+, OrgScreen+ | Static, Deterministic, Aggregate | Not reported |
| Kim[42] | 2013 | USA | Econ | Algorithm | Cyt vs Cyt | >=3y+ | Static, Stochastic, Individual | Formal method |
| Kim[43] | 2005 | UK, Italy, Netherlands, France | Econ | TechIntro | Cyt vs HPV | Cyt_HPV&Cyt>30+, Cyt/HPV+ | Static, Deterministic, Aggregate | Visual inspection |
| Kim[44] | 2002 | USA | Econ | TechIntro | Cyt vs HPV | LBC+, LBC/HPV+ | Static, Deterministic, Aggregate | Unspecified method |
| Knox[45] | 1976 | UK | Epid | Algorithm | Cyt vs Cyt | 25-;30-;(35-80)10x+ | Static, Deterministic, Aggregate | Not reported |
| Koong[46] | 2006 | Taiwan | Econ | ScreenIntro | Cyt vs Cyt | 3y+ | Static, Stochastic, Individual | Not reported |
| Koopmanschap[47] | 1990 | Netherlands | Econ | ScreenIntro | Cyt vs Cyt | OrgPap+, every6y+,37-73 | Static, Stochastic, Individual | Visual inspection |
| Koopmanschap[48] | 1990 | Netherlands | Econ | ScreenIntro | Cyt vs Cyt | OrgPap+, (37-73)6y+ | Static, Stochastic, Individual | Not reported |
| Krahn[49] | 2008 | Canada | Econ | TechIntro | Cyt vs HPV | Pap=LBC, HPVtriage+ | Static, Deterministic, Aggregate | Formal method |
| Kulasingam[50] | 2006 | USA | Econ | TechIntro | Cyt vs HPV | LBC/HPV+ | Static, Deterministic, Aggregate | Not reported |
| Kulasingam[51] | 2006 | USA | Econ | Algorithm | Cyt vs Cyt | (<30)2-3y(>30)3y+ | Static, Deterministic, Aggregate | Not reported |
| Kulasingam[52] | 2009 | Canada | Econ | TechIntro | Cyt vs HPV | HPV/Cyt>25+ | Static, Deterministic, Aggregate | Unspecified method |
| Kulasingam[53] | 2013 | USA | Econ | TechIntro | Cyt vs HPV | Cyt3y_HPV&Cyt5y>30+ | Static, Deterministic, Aggregate | Not reported |
| Lazaar[54] | 2010 | Tunisia | Econ | Algorithm | Cyt vs Cyt | 3y-;5y-;10y+ | Static, Deterministic, Aggregate | Not reported |
| Legood[55] | 2012 | UK | Econ | TechIntro | Cyt vs HPV | HPVtestCure+ | Static, Deterministic, Aggregate | Visual inspection |
| Legood[56] | 2006 | UK | Econ | TechIntro | Cyt vs HPV | LBC&HPV>35+ | Static, Deterministic, Aggregate | Not reported |
| Levin[57] | 2010 | China | Econ | ScreenIntro | Cyt vs HPV | LBC+, CareHPV+ | Static, Deterministic, Aggregate | Unspecified method |
| Mandelblatt[58] | 2002 | Thailand | Econ | ScreenIntro | Cyt vs HPV vs VIA | OrgScreen+, VIA5y(35-55)+,Pap&HPV5y(20-70)+ | Static, Deterministic, Aggregate | Visual inspection |
| Mandelblatt[59] | 1997 | USA | Econ | Algorithm | Cyt vs Cyt | ERscreen+ | Static, Deterministic, Aggregate | not reported |
| Mandelblatt[60] | 2002 | USA | Econ | TechIntro | Cyt vs HPV | HPV&Pap2y+ | Static, Stochastic, Individual | Visual inspection |
| Mandelblatt[61] | 1988 | USA | Econ | Algorithm | Cyt vs Cyt | Screen1x(>65)+ | Static, Deterministic, Aggregate | not reported |
| Mandelblatt[62] | 2004 | USA | Econ | TechIntro | Cyt vs HPV | HPV&Pap2y+ | Static, Deterministic, Aggregate | Not reported |
| Matsunaga[63] | 1997 | Japan | Econ | ScreenIntro | Cyt vs Cyt | Pap+ | Static, Deterministic, Aggregate | Not reported |
| Maxwell[64] | 2002 | USA | Econ | TechIntro | Cyt vs HPV | LBC+, LBC/HPV+ | Static, Deterministic, Aggregate | Not reported |
| McCrory[65] | 1999 | USA | Econ | TechIntro | Cyt vs Auto | LBC+/-Auto3y+ | Static, Deterministic, Aggregate | Visual inspection |
| Melnikow[66] | 2010 | Canada | Econ | TechIntro | Cyt vs HPV | Pap+, LBC-, HPV- | Static, Deterministic, Aggregate | Visual inspection |
| Mittendorf[67] | 2003 | Germany | Econ | TechIntro | Cyt vs HPV | HPV/Colp+, HPV&Cyt+ | Static, Deterministic, Aggregate | Not reported |
| Montz[68] | 2001 | USA | Econ | TechIntro | Cyt vs Cyt | LBC+ | Static, Deterministic, Aggregate | Not reported |
| Myers[69] | 2000 | USA | Epid | Algorithm | Cyt vs Cyt | n.a. | Static, Deterministic, Aggregate | Not reported |
| Myers[70] | 2000 | USA | Econ | Algorithm | Cyt vs Cyt | IncFreq-; IncSE&SP+ | Static, Deterministic, Aggregate | Not reported |
| Neville[71] | 2005 | Australia | Econ | TechIntro | Cyt vs Cyt | LBC+ | Static, Deterministic, Aggregate | Not reported |
| Novoa-Vazquez[72] | 2004 | Portugal | Econ | ScreenIntro | Cyt vs Cyt | LBC+, OrgScreen+ | Static, Deterministic, Aggregate | Not reported |
| Ostensson[73] | 2010 | Sweden | Econ | TechIntro | Cyt vs HPV | Pap/HPV+ | Static, Deterministic, Aggregate | Not reported |
| Ostensson[74] | 2013 | Sweden | Econ | TechIntro | Cyt vs SS | SS(>35)5y+, ConvCyt<35+ | Static, Deterministic, Aggregate | Visual inspection |
| Parkin[75] | 1985 | UK | Epid | Algorithm | Cyt vs Cyt | Screen+ | Static, Stochastic, Individual | Not reported |
| Parkin[76] | 1986 | UK | Econ | Algorithm | Cyt vs Cyt | <35-;(>35)5y+ | Static, Stochastic, Individual | Not reported |
| Perkins[77] | 2010 | Honduras | Econ | ScreenIntro | Cyt vs VIA | VIA+, Pap- | Static, Deterministic, Aggregate | Not reported |
| Philips[78] | 2001 | UK | Econ | TechIntro | Cyt vs HPV | Pap = Pap/HPV; EarlyWithdrawal- | Static, Deterministic, Aggregate | Unspecified method |
| Raab[79] | 1999 | USA | Econ | TechIntro | Cyt vs Cyt | Pap+, newTech- | Static, Deterministic, Aggregate | Not reported |
| Raab[80] | 1997 | USA | Econ | Algorithm | Cyt vs Cyt | PapRescreen+/- | Static, Deterministic, Aggregate | Not reported |
| Raab[81] | 1999 | USA | Econ | Algorithm | Cyt vs Cyt | PapRescreenHigh-risk+ | Static, Deterministic, Aggregate | Not reported |
| Raab[82] | 1998 | USA | Econ | Algorithm | Cyt vs Cyt | ASCUS: Colp-,Treat-, Pap1y+ | Static, Deterministic, Aggregate | Not reported |
| Radensky[83] | 1998 | USA | Econ | TechIntro | Cyt vs Auto | Auto+ | Static, Deterministic, Aggregate | Not reported |
| Raffle[84] | 2003 | UK | Epid | Algorithm | Cyt vs Cyt | SceenIntro+ | Static, Deterministic, Aggregate | Not reported |
| Sato[85] | 1999 | Japan | Econ | ScreenIntro | Cyt vs Cyt | ScreenIntro+ | Static, Deterministic, Aggregate | Not reported |
| Sawaya[86] | 2003 | USA | Epid | Algorithm | Cyt vs Cyt | 30-64:1-3y-;1-1-3y-;1-1-1-3y+ | Static, Deterministic, Aggregate | Not reported |
| Schechter[87] | 1996 | USA | Econ | TechIntro | Cyt vs Auto | Auto+,2y+ | Static, Deterministic, Aggregate | Not reported |
| Sheriff[88] | 2007 | Germany | Econ | TechIntro | Cyt vs HPV | Pap/HPV+, Pap/Pap-, Pap/Colp- | Static, Deterministic, Aggregate | Not reported |
| Sherlaw-Johnson[89] | 2004 | UK | Econ | TechIntro | Cyt vs HPV | LBC+, HPV&LBC5y+ | Static, Deterministic, Aggregate | Not reported |
| Sherlaw-Johnson[90] | 2000 | Eastern Europe | Econ | TechIntro | Cyt vs HPV | HPV/Colp+ | Static, Stochastic, Individual | Unspecified method |
| Sherlaw-Johnson[91] | 1997 | Developing countries | Econ | ScreenIntro | Cyt vs HPV | HPV1x(30-59)+ | Static, Stochastic, Individual | Unspecified method |
| Sherlaw-Johnson[92] | 1999 | UK | Econ | Algorithm | Cyt vs HPV | Stop50,55,60-;65+, HPV&Cyt=Cyt | Static, Stochastic, Individual | Unspecified method |
| Sherlaw-Johnson[93] | 1994 | Latin America, Finland, Iceland | Epid | Algorithm | Cyt vs Cyt | ASCUS:Colp-Cyt6mth+;IncCov+ | Static, Stochastic, Individual | Not reported |
| Shi[94] | 2011 | China | Econ | ScreenIntro | HPV vs VIA | CareHPV+, VIA- | Dynamic, Deterministic, Aggregate | Not reported |
| Shun-Zhang[95] | 1982 | Canada | Epid | ScreenIntro | Cyt vs Cyt | Screen+; Pap(25-52)3y OR (25-40)3y+(40-60)5y+ | Static, Deterministic, Aggregate | Not reported |
| Siebert[96] | 2006 | Germany | Epid | Algorithm | Cyt vs Cyt | 2y+ | Static, Deterministic, Aggregate | Visual inspection |
| Smith[97] | 1999 | USA | Econ | TechIntro | Cyt vs Auto | Auto+ | Static, Deterministic, Aggregate | Not reported |
| Sroczynski[98] | 2011 | Germany | Econ | TechIntro | Cyt vs HPV | HPV/Cyt2yOlder+ | Static, Deterministic, Aggregate | Visual inspection |
| Stout[99] | 2008 | USA | Epid | TechIntro | Cyt vs HPV | LBC/HPV+_HPV/LBC(>30)+ | Static, Stochastic, Individual | Formal method |
| Straughn[100] | 2004 | USA | Econ | TechIntro | Cyt vs HPV | LBC/HPV2y+ | Static, Deterministic, Aggregate | Not reported |
| Suba[101] | 2001 | Vietnam | Econ | ScreenIntro | Cyt vs Cyt | ScreenIntro+ | Static, Deterministic, Aggregate | Not reported |
| Taylor[102] | 2000 | USA | Econ | TechIntro | Conv+/-Spec | 2y+ | Static, Deterministic, Aggregate | Not reported |
| van Ballegooijen[103] | 2000 | UK, Italy, Netherlands, Germany, France, Spain, Portugal, Greece, Denmark, Finland, Sweden, Belgium, Ireland | Epid | Algorithm | Cyt vs Cyt | n.a. | Static, Stochastic, Individual | Not reported |
| van Ballegooijen[104] | 1992 | Netherlands | Epid | ScreenIntro | Cyt vs Cyt | OrgScreen+,Pap(30-60)5y+ | Static, Stochastic, Individual | Not reported |
| van Ballegooijen[105] | 1997 | Netherlands | Econ | TechIntro | Cyt vs HPV | HPV&Pap = Pap | Static, Stochastic, Individual | Not reported |
| van den Akker-van Marle[106] | 2002 | Netherlands | Econ | Algorithm | Cyt vs Cyt | >=3y+ | Static, Stochastic, Individual | Visual inspection |
| van Oortmarssen[107] | 1992 | Canada | Epid | Algorithm | Cyt vs Cyt | Pap(35-64)5y+ | Static, Deterministic, Aggregate | Unspecified method |
| van Rosmalen[108] | 2012 | Netherlands | Econ | TechIntro | Cyt vs HPV | LBC-, Pap_HPV/Pap(>32)+ | Static, Stochastic, Individual | Visual inspection |
| Vanni[109] | 2011 | Brazil | Econ | TechIntro | Cyt vs HPV | HPV/Cyt1y+ | Static, Deterministic, Aggregate | Formal method |
| Vanni[110] | 2011 | Brazil | Econ | TechIntro | Cyt vs HPV | Cyt/HPV(>30)+ | Static, Deterministic, Aggregate | Formal method |
| Vijayaraghavan[111] | 2009 | South Africa | Econ | TechIntro | Cyt vs HPV | HPV/Pap+ | Static, Deterministic, Aggregate | Not reported |
| Vijayaraghavan[112] | 2010 | Canada | Econ | TechIntro | Cyt vs HPV | Pap-, Pap/HPV+, HPV/Pap+, HPV&Pap+ | Static, Deterministic, Aggregate | Not reported |
| Vijayaraghavan[113] | 2010 | USA | Econ | TechIntro | Cyt vs HPV vs GT | HPV&LBC/GT+ | Static, Deterministic, Aggregate | Visual inspection |
| Voko[114] | 2012 | Hungary | Econ | Algorithm | Cyt vs Cyt | Improve Cov+ | Static, Deterministic, Aggregate | Visual inspection |
| Willis[115] | 2005 | UK | Epid | TechIntro | Cyt vs Auto | Cyt+, Auto- | Static, Stochastic, Individual | Not reported |
| Woo[116] | 2007 | China | Econ | ScreenIntro | Cyt vs Cyt | 4y+ | Static, Deterministic, Aggregate | Not reported |
| Woo[117] | 2005 | Hong Kong | Epid | ScreenIntro | Cyt vs Cyt | OrgScreen+ | Static, Deterministic, Aggregate | Visual inspection |
| Wu[118] | 2011 | Hong Kong | Econ | TechIntro | Cyt vs HPV | HPV&Cyt3y+ | Static, Stochastic, Individual | Visual inspection |

# References

1. Agorastos T, Sotiriadis A, Emmanouilides CJ. Effect of type-specific human papillomavirus incidence on screening performance and cost [Internet]. International journal of gynecological cancer : official journal of the International Gynecological Cancer Society. United States: First Department of Obstetrics and Gynecology, Papageorgiou General Hospital, Aristotle University of Thessaloniki, Thessaloniki, Greece. agorast@auth.gr; 2010. p. 276–82. Available from: http://ovidsp.ovid.com/ovidweb.cgi?T=JS&PAGE=reference&D=emed9&NEWS=N&AN=20134270

2. Anderson R, Haas M, Shanahan M. The cost-effectiveness of cervical screening in Australia: what is the impact of screening at different intervals or over a different age range? Aust N Z J Public Health [Internet]. 2008;32(1):43–52. Available from: http://onlinelibrary.wiley.com/o/cochrane/cleed/articles/NHSEED-22008000624/frame.html

3. Andres-Gamboa O, Chicaiza L, Garcia-Molina M, Diaz J, Gonzalez M, Murillo R, et al. Cost-effectiveness of conventional cytology and HPV DNA testing for cervical cancer screening in Colombia. [Internet]. Salud publica de Mexico. Mexico: Clinical Research Group, National Cancer Institute of Colombia, Bogota Colombia. ogamboa@cancer.gov.co; 2008. p. 276–85. Available from: http://ovidsp.ovid.com/ovidweb.cgi?T=JS&PAGE=reference&D=med4&NEWS=N&AN=18670718

4. Atashili J, Smith JS, Adimora AA, Eron J, Miller WC, Myers E. Potential impact of antiretroviral therapy and screening on cervical cancer mortality in HIV-positive women in sub-Saharan Africa: a simulation. [Internet]. PloS one. United States: Faculty of Health Sciences, University of Buea, Buea, Cameroon. atashili@yahoo.ie; 2011. p. e18527. Available from: http://ovidsp.ovid.com/ovidweb.cgi?T=JS&PAGE=reference&D=medl&NEWS=N&AN=21483701

5. Balasubramanian A, Kulasingam SL, Baer A, Hughes JP, Myers ER, Mao C, et al. Accuracy and cost-effectiveness of cervical cancer screening by high-risk human papillomavirus DNA testing of self-collected vaginal samples. [Internet]. Journal of lower genital tract disease. United States: Department of Epidemiology, University of Washington, Seattle, WA, USA. akhilab@amgen.com; 2010. p. 185–95. Available from: http://ovidsp.ovid.com/ovidweb.cgi?T=JS&PAGE=reference&D=medc&NEWS=N&AN=20592553

6. Berkhof J, Bruijne MC, Zielinski GD, Bulkmans NW, Rozendaal L, Snijders PJ, et al. Evaluation of cervical screening strategies with adjunct high-risk human papillomavirus testing for women with borderline or mild dyskaryosis [Internet]. International Journal of Cancer. J. Berkhof, Department of Clinical Epidemiology and Biostatistics, VU University Medical Centre, PO Box 7057, 1007 MB, Amsterdam, Netherlands. E-mail: h.berkhof@vumc.nl: Wiley-Liss Inc.; 2006. p. 1759–68. Available from: http://ovidsp.ovid.com/ovidweb.cgi?T=JS&PAGE=reference&D=emed7&NEWS=N&AN=2006121460

7. Berkhof J, Coupe VM, Bogaards JA, van Kemenade FJ, Helmerhorst TJ, Snijders PJ, et al. The health and economic effects of HPV DNA screening in The Netherlands. [Internet]. International journal of cancer. Journal international du cancer. United States: Department of Epidemiology and Biostatistics, VU University Medical Centre, Amsterdam, The Netherlands.; 2010. p. 2147–58. Available from: http://ovidsp.ovid.com/ovidweb.cgi?T=JS&PAGE=reference&D=medl&NEWS=N&AN=20112339

8. Bidus MA, Maxwell GL, Kulasingam S, Rose GS, Elkas JC, Chernofsky M, et al. Cost-effectiveness analysis of liquid-based cytology and human papillomavirus testing in cervical cancer screening. [Internet]. Obstetrics and gynecology. United States: Division of Gynecologic Oncology and the United States Military Cancer Institute, Walter Reed Army Medical Center, Washington, DC 20307, USA.; 2006. p. 997–1005. Available from: http://ovidsp.ovid.com/ovidweb.cgi?T=JS&PAGE=reference&D=med4&NEWS=N&AN=16648402

9. Bistoletti P, Sennfalt K, Dillner J. Cost-effectiveness of primary cytology and HPV DNA cervical screening [Internet]. International Journal of Cancer. United States: Department of Obstetrics and Gynecology, Nacka Hospital, Nacka, Sweden.; 2008. p. 372–6. Available from: http://onlinelibrary.wiley.com/o/cochrane/cleed/articles/NHSEED-22008000026/frame.html

10. Boyd A, Davies LA, Bagust A. Modelling the implications for hospital services of cervical cytology screening: a case history [Internet]. The Journal of the Operational Research Society. A. Boyd; 1989. p. 529–37. Available from: http://ovidsp.ovid.com/ovidweb.cgi?T=JS&PAGE=reference&D=emed2&NEWS=N&AN=10293891

11. Brown AD, Garber AM. Cost-effectiveness of 3 methods to enhance the sensitivity of papanicolaou testing. J Am Med Assoc [Internet]. UNITED STATES: Department of Public Health and Primary Care, University of Oxford, England.; 1999;281(4):347–53. Available from: http://onlinelibrary.wiley.com/o/cochrane/cleed/articles/NHSEED-21999008015/frame.html

12. Burger EA, Ortendahl JD, Sy S, Kristiansen IS, Kim JJ. Cost-effectiveness of cervical cancer screening with primary human papillomavirus testing in Norway. [Internet]. British journal of cancer. England: Department of Health Management and Health Economics, University of Oslo, Norway.; 2012. p. 1571–8. Available from: http://ovidsp.ovid.com/ovidweb.cgi?T=JS&PAGE=reference&D=medc&NEWS=N&AN=22441643

13. Campos NG, Castle PE, Schiffman M, Kim JJ. Policy implications of adjusting randomized trial data for economic evaluations: a demonstration from the ASCUS-LSIL Triage Study. [Internet]. Medical decision making : an international journal of the Society for Medical Decision Making. United States: Center for Health Decision Science, Harvard School of Public Health, Boston, MA, USA. gastin@post.harvard.edu; 2012. p. 400–27. Available from: http://ovidsp.ovid.com/ovidweb.cgi?T=JS&PAGE=reference&D=medc&NEWS=N&AN=22147881

14. Canfell K, Barnabas R, Patnick J, Beral V. The predicted effect of changes in cervical screening practice in the UK: results from a modelling study [Internet]. British journal of cancer. England: Cancer Research UK Epidemiology Unit, University of Oxford, Gibson Building, Radcliffe Infirmary, OX2 6HE, UK.; 2004. p. 530–6. Available from: http://ovidsp.ovid.com/ovidweb.cgi?T=JS&PAGE=reference&D=med4&NEWS=N&AN=15266332

15. Chow IH-I, Tang C-H, You S-L, Liao C-H, Chu T-Y, Chen C-JC-AC-JC-A, et al. Cost-effectiveness analysis of human papillomavirus DNA testing and Pap smear for cervical cancer screening in a publicly financed health-care system. [Internet]. British journal of cancer. England: School of Health Care Administration, Taipei Medical University, Taipei, Taiwan.; 2010. p. 1773–82. Available from: http://ovidsp.ovid.com/ovidweb.cgi?T=JS&PAGE=reference&D=medl&NEWS=N&AN=21102588

16. Chuck A. Cost-effectiveness of 21 alternative cervical cancer screening strategies. [Internet]. Value in health : the journal of the International Society for Pharmacoeconomics and Outcomes Research. United States: Institute of Health Economics-Decision Analytic Modeling Unit, Edmonton, Alberta, Canada. achuck@ihe.ca; 2010. p. 169–79. Available from: http://ovidsp.ovid.com/ovidweb.cgi?T=JS&PAGE=reference&D=medl&NEWS=N&AN=19804436

17. Coppleson LW, Brown B. The prevention of carcinoma of the cervix. Am J Obstet Gynecol. UNITED STATES; 1976;125:153–9.

18. Coupe VMH, Berkhof J, Verheijen RHM, Meijer CJLM. Cost-effectiveness of human papillomavirus testing after treatment for cervical intraepithelial neoplasia [Internet]. BJOG : an international journal of obstetrics and gynaecology. England: Department of Clinical Epidemiology and Biostatistics, VU University Medical Centre, Amsterdam, The Netherlands. v.coupe@vumc.nl; 2007. p. 416–24. Available from: http://ovidsp.ovid.com/ovidweb.cgi?T=JS&PAGE=reference&D=med4&NEWS=N&AN=17378816

19. Creighton P, Lew J-BB, Clements M, Smith MA, Howard K, Dyer S, et al. Cervical cancer screening in Australia: modelled evaluation of the impact of changing the recommended interval from two to three years [Internet]. BMC public health. P. Creighton, Cancer Epidemiology Research Unit, Cancer Council NSW, Sydney, Australia.: Cancer Epidemiology Research Unit, Cancer Council NSW, Sydney, Australia.; 2010. p. 734. Available from: http://ovidsp.ovid.com/ovidweb.cgi?T=JS&PAGE=reference&D=medl&NEWS=N&AN=21110881

20. De Bekker-Grob EW, de Kok IMCM, Bulten J, van Rosmalen J, Vedder JEM, Arbyn M, et al. Liquid-based cervical cytology using ThinPrep technology: weighing the pros and cons in a cost-effectiveness analysis. [Internet]. Cancer causes & control : CCC. Netherlands: Department of Public Health, Erasmus MC-University Medical Centre Rotterdam, Rotterdam, The Netherlands. e.debekker@erasmusmc.nl; 2012. p. 1323–31. Available from: http://ovidsp.ovid.com/ovidweb.cgi?T=JS&PAGE=reference&D=medl&NEWS=N&AN=22706692

21. De Kok IMCMIMCM, van Rosmalen J, Dillner J, Arbyn M, Sasieni P, Iftner T, et al. Primary screening for human papillomavirus compared with cytology screening for cervical cancer in European settings: cost effectiveness analysis based on a Dutch microsimulation model. BMJ [Internet]. I.M. de Kok, Erasmus MC, University Medical Center, Department of Public Health, PO Box 2040, 3000 CA Rotterdam, Netherlands.: Erasmus MC, University Medical Center, Department of Public Health, PO Box 2040, 3000 CA Rotterdam, Netherlands. i.dekok@erasmusmc.nl; 2012;344:e670. Available from: http://ovidsp.ovid.com/ovidweb.cgi?T=JS&PAGE=reference&D=medl&NEWS=N&AN=22391612

22. Dewilde S, Anderson R. The cost-effectiveness of screening programs using single and multiple birth cohort simulations: a comparison using a model of cervical cancer. Med Decis Making [Internet]. United States: MEDTAP International Inc., London, UK.; 2004;24(5):486–92. Available from: http://ovidsp.ovid.com/ovidweb.cgi?T=JS&PAGE=reference&D=med4&NEWS=N&AN=15358997

23. Eddy DM. The frequency of cervical cancer screening. Comparison of a mathematical model with empirical data. [Internet]. Cancer. UNITED STATES; 1987. p. 1117–22. Available from: http://ovidsp.ovid.com/ovidweb.cgi?T=JS&PAGE=reference&D=med2&NEWS=N&AN=3607728

24. Fennessy P, Raulli A, Abdulwadud O, Petherick E, Bryan E, Harris C, et al. Liquid based cytology for cervical screening. Database Abstr Rev Eff [Internet]. Medical Services Advisory Committee; 2002;(2):97. Available from: http://onlinelibrary.wiley.com/o/cochrane/cldare/articles/DARE-12004008041/frame.html

25. Fetters MD, Lieberman RW, Abrahamse PH, Sanghvi R V, Sonnad SS. Cost-effectiveness of pap smear screening for vaginal cancer after total hysterectomy for benign disease. J Low Genit Tract Dis [Internet]. 2003 Jul;7(3):194–202. Available from: http://www.ncbi.nlm.nih.gov/pubmed/17051068

26. Flores YN, Bishai DM, Lorincz A, Shah K V, Lazcano-Ponce E, Hernandez M, et al. HPV testing for cervical cancer screening appears more cost-effective than Papanicolau cytology in Mexico. [Internet]. Cancer causes & control : CCC. Netherlands: Instituto Mexicano del Seguro Social, Cuernavaca, Morelos, Mexico. yflores@jhsph.edu; 2011. p. 261–72. Available from: http://ovidsp.ovid.com/ovidweb.cgi?T=JS&PAGE=reference&D=medl&NEWS=N&AN=21170578

27. Frame PS, Frame JS. Determinants of cancer screening frequency: the example of screening for cervical cancer [Internet]. The Journal of the American Board of Family Practice / American Board of Family Practice. P.S. Frame, Tri-County Family Medicine Program, Cohocton, NY, USA.: Tri-County Family Medicine Program, Cohocton, NY, USA.; 1998. p. 87–95. Available from: http://ovidsp.ovid.com/ovidweb.cgi?T=JS&PAGE=reference&D=emed4&NEWS=N&AN=9542700

28. Goldie SJ, Kim JJ, Wright TC. Cost-effectiveness of human papillomavirus DNA testing for cervical cancer screening in women aged 30 years or more. [Internet]. Obstetrics and gynecology United States: Lippincott Williams and Wilkins; 2004 p. 619–31. Available from: http://ovidsp.ovid.com/ovidweb.cgi?T=JS&PAGE=reference&D=med4&NEWS=N&AN=15051550

29. Goldie SJ, Freedberg KA, Weinstein MC, Wright TC, Kuntz KM. Cost effectiveness of human papillomavirus testing to augment cervical cancer screening in women infected with the human immunodeficiency virus. Am J Med [Internet]. United States: Harvard Center for Risk Analysis, Department of Health Policy and Management, Harvard School of Public Health, Boston, Massachusetts 02115-5924, USA.; 2001;111(2):140–9. Available from: http://ovidsp.ovid.com/ovidweb.cgi?T=JS&PAGE=reference&D=emed5&NEWS=N&AN=2001279775

30. Goldie SJ, Gaffikin L, Goldhaber-Fiebert JD, Gordillo-Tobar A, Levin CE, Mahe C, et al. Cost-effectiveness of cervical-cancer screening in five developing countries [Internet]. New England Journal of Medicine. United States: Department of Health Policy and Management, Harvard School of Public Health, Boston, USA. sue_goldie@harvard.edu; 2005. p. 2158–68. Available from: http://content.nejm.org/cgi/reprint/353/20/2158.pdf

31. Goldie SJ, Kuhn L, Denny L, Pollack A, Wright TC. Policy analysis of cervical cancer screening strategies in low-resource settings: clinical benefits and cost-effectiveness. JAMA [Internet]. United States: Center for Risk Analysis, Department of Health Policy and Management, Harvard School of Public Health, 718 Huntington Ave, Suite 2, Boston, MA 02115-5924, USA. sgoldie@hsph.harvard.edu; 2001;285(24):3107–15. Available from: http://onlinelibrary.wiley.com/o/cochrane/cleed/articles/NHSEED-22001008233/frame.html

32. Goldie SJ, Weinstein MC, Kuntz KM, Freedberg KA. The costs, clinical benefits, and cost-effectiveness of screening for cervical cancer in HIV-infected women. Ann Intern Med. 1999;130:97-107.

33. Gustafsson L, Adami HO. Optimization of cervical cancer screening. [Internet]. Cancer causes & control : CCC. ENGLAND: Department of Technology, Uppsala University, Sweden.; 1992. p. 125–36. Available from: http://ovidsp.ovid.com/ovidweb.cgi?T=JS&PAGE=reference&D=med3&NEWS=N&AN=1562702

34. Gutierrez-Aguado A. [Cost-utility of the vaccine against the Human Papiloma Virus in Peruvian women]. [Internet]. Costo-utilidad de la vacuna contra el Virus de Papiloma Humano en mujeres peruanas. Peru: Facultad de Ciencias Medicas, Universidad Cesar Vallejo-Lima Norte, Lima, Peru. agutierreza@ucvlima.edu.pe; 2011. p. 416–25. Available from: http://ovidsp.ovid.com/ovidweb.cgi?T=JS&PAGE=reference&D=medl&NEWS=N&AN=22086620

35. Gyrd-Hansen D, Holund B, Andersen P. A cost-effectiveness analysis of cervical cancer screening: health policy implications. NHS Econ Eval Database [Internet]. IRELAND: Center for Health and Social Policy, Odense University, Denmark.; 1995;34(1):35–51. Available from: http://ovidsp.ovid.com/ovidweb.cgi?T=JS&PAGE=reference&D=med3&NEWS=N&AN=10151964

36. Hadwin R, Eggington S, Brennan A, Walker P, Patnick J, Pilgrim H. Modelling the cost-effectiveness and capacity impact of changes to colposcopy referral guidelines for women with mild dyskaryosis in the UK Cervical Screening Programme. [Internet]. BJOG. An International Journal of Obstetrics and Gynaecology. England: Department of Colposcopy, Royal Free Hospital, London, UK. richard.hadwin@yahoo.co.uk; 2008. p. 749–57. Available from: http://ovidsp.ovid.com/ovidweb.cgi?T=JS&PAGE=reference&D=med4&NEWS=N&AN=18410660

37. Helfand M, O’Connor GT, Zimmer-Gembeck M, Beck JR. Effect of the Clinical Laboratory Improvement Amendments of 1988 (CLIA ’88) on the incidence of invasive cervical cancer. Med Care [Internet]. UNITED STATES: Division of General Internal Medicine, Portland VA Medical Center, OR 97207.; 1992;30(12):1067–82. Available from: http://ovidsp.ovid.com/ovidweb.cgi?T=JS&PAGE=reference&D=med3&NEWS=N&AN=1453813

38. Hughes AA, Glazner J, Barton P, Shlay JC. A cost-effectiveness analysis of four management strategies in the determination and follow-up of atypical squamous cells of undetermined significance. Diagn Cytopathol [Internet]. United States: Department of Preventive Medicine, University of Colorado Health Sciences Center, Denver, Colorado 80204, USA. Alice.Hughes@rmpdc.org; 2005;32(2):125–32. Available from: http://ovidsp.ovid.com/ovidweb.cgi?T=JS&PAGE=reference&D=med4&NEWS=N&AN=15637677

39. Hutchinson ML, Berger BM, Farber FL. Clinical and cost implications of new technologies for cervical cancer screening: the impact of test sensitivity [Internet]. The American journal of managed care. UNITED STATES: Department of Pathology and Laboratory Medicine, Woimen and Infants’ Hospital of Rhode Island, Providence, USA. mhutchin@earthlink.net; 2000. p. 766–80. Available from: http://ovidsp.ovid.com/ovidweb.cgi?T=JS&PAGE=reference&D=med4&NEWS=N&AN=11067374

40. Karnon J, Peters J, Platt J, Chilcott J, McGoogan E, Brewer N. Liquid-based cytology in cervical screening: an updated rapid and systematic review and economic analysis. [Internet]. Health technology assessment (Winchester, England). England: The School of Health and Related Research The University of Sheffield, Sheffield, UK.; 2004. p. 1–90. Available from: http://ovidsp.ovid.com/ovidweb.cgi?T=JS&PAGE=reference&D=emed6&NEWS=N&AN=2004269297

41. Kim JJ, Leung GM, Woo PPS, Goldie SJ. Cost-effectiveness of organized versus opportunistic cervical cytology screening in Hong Kong. J Public Health (Oxf) [Internet]. England: Harvard Center for Risk Analysis, Department of Health Policy and Management, Harvard School of Public Health, Boston, MA, USA.; 2004;26(2):130–7. Available from: http://onlinelibrary.wiley.com/o/cochrane/cleed/articles/NHSEED-22004008266/frame.html

42. Kim JJ, Sharma M, Ortendahl J. Optimal interval for routine cytologic screening in the United States. JAMA Intern Med [Internet]. United States: American Medical Association (515 North State Street, Chicago IL 60654, United States); 2013;173(3):241–2. Available from: http://archinte.jamanetwork.com/data/Journals/INTEMED/926380/ilt120022_241_242.pdf

43. Kim JJ, Wright TC, Goldie SJ. Cost-effectiveness of human papillomavirus DNA testing in the United Kingdom, The Netherlands, France, and Italy. J Natl Cancer Inst [Internet]. United States: Department of Health Policy and Management, Harvard School of Public Health, Boston, MA, USA.; 2005;97(12):888–95. Available from: http://ovidsp.ovid.com/ovidweb.cgi?T=JS&PAGE=reference&D=med4&NEWS=N&AN=15956650

44. Kim JJ, Wright TC, Goldie SJ. Cost-effectiveness of alternative triage strategies for atypical squamous cells of undetermined significance. [Internet]. JAMA : the journal of the American Medical Association. United States: Department of Health Policy and Management, Harvard Center for Risk Analysis, 718 Huntington Ave, Second Floor, Boston, MA 02115, USA.; 2002. p. 2382–90. Available from: http://ovidsp.ovid.com/ovidweb.cgi?T=JS&PAGE=reference&D=med4&NEWS=N&AN=11988059

45. Knox EG. Ages and frequencies for cervical cancer screening. [Internet]. British journal of cancer. ENGLAND; 1976. p. 444–52. Available from: http://ovidsp.ovid.com/ovidweb.cgi?T=JS&PAGE=reference&D=med1&NEWS=N&AN=823954

46. Koong S-L, Yen AM-F, Chen TH-H. Efficacy and cost-effectiveness of nationwide cervical cancer screening in Taiwan. J Med Screen [Internet]. England: Cancer Control and Prevention Division, Bureau of Health Promotion, Department of Health, Sinjhuang City, Taipei County 242, Taiwan.; 2006;13(suppl 1:S44–7. Available from: http://ovidsp.ovid.com/ovidweb.cgi?T=JS&PAGE=reference&D=med4&NEWS=N&AN=17227642

47. Koopmanschap MA, Lubbe KT, Oortmarssen GJ, Agt HM, Ballegooijen M, Habbema JD. Economic aspects of cervical cancer screening. Soc Sci Med [Internet]. 1990;30(10):1081–7. Available from: http://onlinelibrary.wiley.com/o/cochrane/cleed/articles/NHSEED-21995005287/frame.html

48. Koopmanschap MA, van Oortmarssen GJ, van Agt HM, van Ballegooijen M, Habbema JD, Lubbe KT. Cervical-cancer screening: attendance and cost-effectiveness. Int J Cancer [Internet]. UNITED STATES: Department of Public Health and Social Medicine, Erasmus University, Rotterdam, The Netherlands.; 1990;45(3):410–5. Available from: http://ovidsp.ovid.com/ovidweb.cgi?T=JS&PAGE=reference&D=med3&NEWS=N&AN=2106499

49. Krahn M, McLachlin M, Pham B, Rosen B, Sander B, Grootendorst P, et al. Liquid-based techniques for cervical cancer screening: systematic review and cost-effectiveness analysis. Heal Technol Assess Database. Canadian Agency for Drugs and Technologies in Health (CADTH); 2008;79.

50. Kulasingam SL, Kim JJ, Lawrence WF, Mandelblatt JS, Myers ER, Schiffman M, et al. Cost-effectiveness analysis based on the atypical squamous cells of undetermined significance/low-grade squamous intraepithelial lesion Triage Study (ALTS). [Internet]. Journal of the National Cancer Institute. United States: Department of Obstetrics and Gynecology, Duke University, Durham, NC, USA. kulas002@mc.duke.edu; 2006. p. 92–100. Available from: http://ovidsp.ovid.com/ovidweb.cgi?T=JS&PAGE=reference&D=med4&NEWS=N&AN=16418511

51. Kulasingam SL, Myers ER, Lawson HW, McConnell KJ, Kerlikowske K, Melnikow J, et al. Cost-effectiveness of extending cervical cancer screening intervals among women with prior normal pap tests. Obstet Gynecol [Internet]. United States: Department of Obstetrics, Gynecology and Reproductive Sciences, University of California, San Francisco, USA. kulas002@mc.duke.edu; 2006;107(2 Pt 1):321–8. Available from: http://ovidsp.ovid.com/ovidweb.cgi?T=JS&PAGE=reference&D=med4&NEWS=N&AN=16449119

52. Kulasingam SL, Rajan R, Pierre Y, Atwood C V, Myers ER, Franco EL. Human papillomavirus testing with Pap triage for cervical cancer prevention in Canada: a cost-effectiveness analysis. BMC Med [Internet]. 2009;7:69(2). Available from: http://onlinelibrary.wiley.com/o/cochrane/cleed/articles/NHSEED-22010000265/frame.html

53. Kulasingam SL, Havrilesky LJ, Ghebre R, Myers ER. Screening for cervical cancer: A modeling study for the us preventive services task force. J Low Genit Tract Dis [Internet]. S.L. Kulasingam, Division of Epidemiology and Community Health, University of Minnesota, School of Public Health, 1300 South 2nd St, Minneapolis, MN 55454, United States. E-mail: kulas016@umn.edu: Lippincott Williams and Wilkins (530 Walnut Street,P O Box 327, Philadelphia PA 19106-3621, United States); 2013;17(2):193–202. Available from: http://ovidsp.ovid.com/ovidweb.cgi?T=JS&PAGE=reference&D=emed11&NEWS=N&AN=2013233509

54. Lazaar HBG, Aounallah-Skhiri H, Oueslati F, Frikha H, Achour N, Hsairi M. [Cost effectiveness analysis of screening strategies for cervical cancer in Tunisia]. Analyse cout-efficacite des strategies de depistage du cancer du col uterin en Tunisie. East Mediterr Heal J [Internet]. Egypt: Institut national de la Sante publique, Tunis, Tunisie.; 2010;16(6):602–8. Available from: http://ovidsp.ovid.com/ovidweb.cgi?T=JS&PAGE=reference&D=medl&NEWS=N&AN=20799586

55. Legood R, Smith MA, Lew J-BB, Walker R, Moss S, Kitchener HHC, et al. Cost effectiveness of human papillomavirus test of cure after treatment for cervical intraepithelial neoplasia in England: Economic analysis from NHS Sentinel Sites Study [Internet]. BMJ (Online) R. Legood, Health Services Research and Policy Unit, London School of Hygiene and Tropical Medicine, London WC1 9HS, United Kingdom. E-mail: rosa.legood@lshtm.ac.uk: BMJ Publishing Group (Tavistock Square, London WC1H 9JR, United Kingdom); 2012 p. e7086. Available from: http://ovidsp.ovid.com/ovidweb.cgi?T=JS&PAGE=reference&D=medc&NEWS=N&AN=23117060

56. Legood R, Gray A, Wolstenholme J, Moss S. Lifetime effects, costs, and cost effectiveness of testing for human papillomavirus to manage low grade cytological abnormalities: results of the NHS pilot studies. Bmj [Internet]. 2006;332(2):79–85. Available from: http://onlinelibrary.wiley.com/o/cochrane/cleed/articles/NHSEED-22006008050/frame.html

57. Levin CE, Sellors J, Shi J-F, Ma L, Qiao Y, Ortendahl J, et al. Cost-effectiveness analysis of cervical cancer prevention based on a rapid human papillomavirus screening test in a high-risk region of China. [Internet]. International journal of cancer. Journal international du cancer. United States: PATH, Seattle, WA 98107, USA. clevin@path.org; 2010. p. 1404–11. Available from: http://ovidsp.ovid.com/ovidweb.cgi?T=JS&PAGE=reference&D=medl&NEWS=N&AN=20049838

58. Mandelblatt JS, Lawrence WF, Gaffikin L, Limpahayom KK, Lumbiganon P, Warakamin S, et al. Costs and benefits of different strategies to screen for cervical cancer in less-developed countries. J Natl Cancer Inst [Internet]. United States: Department of Oncology, Georgetown University Medical Center, and the Outcomes Core and Cancer Control Program, Lombardi Cancer Center, Washington, DC 20007, USA. mandelbj@gunet.georgetown.edu; 2002;94(19):1469–83. Available from: http://ovidsp.ovid.com/ovidweb.cgi?T=JS&PAGE=reference&D=med4&NEWS=N&AN=12359856

59. Mandelblatt J, Freeman H, Winczewski D, Cagney K, Williams S, Trowers R, et al. The costs and effects of cervical and breast cancer screening in a public hospital emergency room. Am J Public Health [Internet]. UNITED STATES: Department of Epidemiology and Biostatistics, Memorial-Sloan-Kettering Cancer Center, New York City, USA.; 1997;87(7):1182–9. Available from: http://ovidsp.ovid.com/ovidweb.cgi?T=JS&PAGE=reference&D=med4&NEWS=N&AN=9240110

60. Mandelblatt JS, Lawrence WF, Womack SM, Jacobson D, Yi B, Hwang Y, et al. Benefits and costs of using HPV testing to screen for cervical cancer. JAMA [Internet]. United States: Lombardi Cancer Center, 2233 Wisconsin Ave NW, Suite 317, Washington, DC 20007. mandelbj@georgetown.edu; 2002;287(18):2372–81. Available from: http://ovidsp.ovid.com/ovidweb.cgi?T=JS&PAGE=reference&D=med4&NEWS=N&AN=11988058

61. Mandelblatt JS, Fahs MC. The cost-effectiveness of cervical cancer screening for low-income elderly women. [Internet]. JAMA : the journal of the American Medical Association. UNITED STATES: Department of Ambulatory Care, City Hospital Center, Elmhurst, NY.; 1988. p. 2409–13. Available from: http://ovidsp.ovid.com/ovidweb.cgi?T=JS&PAGE=reference&D=med3&NEWS=N&AN=3127608

62. Mandelblatt J, Lawrence W, Yi B, King J. The balance of harms, benefits, and costs of screening for cervical cancer in older women: the case for continued screening. [Internet]. Archives of internal medicine. United States: Department of Medicine and Oncology, Georgetown University Medical Center, Lombardi Cancer Center, Washington, DC 20007, USA. mandelbj@georgetown.edu; 2004. p. 245–8. Available from: http://ovidsp.ovid.com/ovidweb.cgi?T=JS&PAGE=reference&D=med4&NEWS=N&AN=14769618

63. Matsunaga G, Tsuji I, Sato S, Fukao A, Hisamichi S, Yajima A. Cost-effective analysis of mass screening for cervical cancer in Japan (Structured abstract) [Internet]. Journal of Epidemiology. JAPAN: Department of Obstetrics and Gynecology, Tohoku University School of Medicine, Sendai, Japan.; 1997. p. 135–41. Available from: http://onlinelibrary.wiley.com/o/cochrane/cleed/articles/NHSEED-21998006108/frame.html

64. Maxwell GL, Carlson JW, Ochoa M, Krivak T, Rose GS, Myers ER. Costs and effectiveness of alternative strategies for cervical cancer screening in military beneficiaries. [Internet]. Obstetrics and Gynecology. United States: Division of Gynecologic Oncology, Walter Reed Army Medical Center, Washington, District of Columbia, USA.; 2002. p. 740–8. Available from: http://ovidsp.ovid.com/ovidweb.cgi?T=JS&PAGE=reference&D=med4&NEWS=N&AN=12383543

65. McCrory DC, Matchar DB, Bastian L, Datta S, Hasselblad V, Hickey J, et al. Evaluation of cervical cytology [Internet]. Database of Abstracts of Reviews of Effects. United States: Evidence Report/Technology Assessment (Summary); 1999. p. 274. Available from: http://ovidsp.ovid.com/ovidweb.cgi?T=JS&PAGE=reference&D=med4&NEWS=N&AN=11925972

66. Melnikow J, Kulasingam S, Slee C, Helms LJ, Kuppermann M, Birch S, et al. Surveillance after treatment for cervical intraepithelial neoplasia: outcomes, costs, and cost-effectiveness. Obstet Gynecol [Internet]. United States: Department of Economics, Center for Healthcare Policy and Research, University of California, Davis, California 95817, USA. jamelnikow@ucdavis.edu; 2010;116(5):1158–70. Available from: http://ovidsp.ovid.com/ovidweb.cgi?T=JS&PAGE=reference&D=medl&NEWS=N&AN=20966702

67. Mittendorf T, Petry KU, Iftner T, Greiner W, von der Schulenburg JM, Schulenburg JM. Economic Evaluation of Human Papillomavirus Screening in Germany. Eur J Heal Econ [Internet]. 2003;4(3):209–15. Available from: http://onlinelibrary.wiley.com/o/cochrane/cleed/articles/NHSEED-22003008275/frame.html

68. Montz FJ, Farber FL, Bristow RE, Cornelison T. Impact of increasing Papanicolaou test sensitivity and compliance: a modeled cost and outcomes analysis. [Internet]. Obstetrics and gynecology. United States: Kelly Gynecologic Oncology Service, The Johns Hopkins Hospital and Medical Institutions, Baltimore, Maryland 21287-1248, USA. fmontz@jhmi.edu; 2001. p. 781–8. Available from: http://ovidsp.ovid.com/ovidweb.cgi?T=JS&PAGE=reference&D=med4&NEWS=N&AN=11339934

69. Myers ER, McCrory DC, Nanda K, Bastian L, Matchar DB. Mathematical model for the natural history of human papillomavirus infection and cervical carcinogenesis. [Internet]. American journal of epidemiology. UNITED STATES: Department of Obstetrics and Gynecology, Duke University Medical Center, Durham, NC 27710, USA. myers008@mc.duke.edu; 2000. p. 1158–71. Available from: http://ovidsp.ovid.com/ovidweb.cgi?T=JS&PAGE=reference&D=med4&NEWS=N&AN=10905528

70. Myers ER, McCrory DC, Subramanian S, McCall N, Nanda K, Datta S, et al. Setting the target for a better cervical screening test: characteristics of a cost-effective test for cervical neoplasia screening. Obstet Gynecol [Internet]. UNITED STATES: Department of Obstetrics and Gynecology, Center for Clinical Health Policy Research-Evidence-Based Practice Center, Durham, North Carolina, USA. myers008@mc.duke.edu; 2000;96(5 Pt 1):645–52. Available from: http://ovidsp.ovid.com/ovidweb.cgi?T=JS&PAGE=reference&D=med4&NEWS=N&AN=11042294

71. Neville AM, Quinn MA. An alternative cost effectiveness analysis of ThinPrep in the Australian setting. [Internet]. The Australian & New Zealand journal of obstetrics & gynaecology. Australia: Pretium, Sydney, Australia. munro.neville@pretium.com.au; 2005. p. 289–94. Available from: http://ovidsp.ovid.com/ovidweb.cgi?T=JS&PAGE=reference&D=med4&NEWS=N&AN=16029294

72. Novoa Vazquez RM. [Cost-effectiveness of a cervical cancer screening programme in the Algarve region, Portugal]. Anal coste-efectividad del programa deteccion Sist del cancer Cerv en la Reg del Algarve, Port [Internet]. Spain: Centro Regional de Saude Publica, Administracion Regional de Saude del Algarve, Faro, Portugal. rosamarianovoa@yahoo.es; 2004;78(3):341–53. Available from: http://ovidsp.ovid.com/ovidweb.cgi?T=JS&PAGE=reference&D=med4&NEWS=N&AN=15293955

73. Ostensson E, Froberg M, Hjerpe A, Zethraeus N, Andersson S. Economic analysis of human papillomavirus triage, repeat cytology, and immediate colposcopy in management of women with minor cytological abnormalities in Sweden. Acta Obstet Gynecol Scand [Internet]. England: Department of Clinical Science, Intervention and Technology (CLINTEC), Division of Obstetrics and Gynecology, Karolinska University Hospital-Huddinge, Karolinska Institutet, Stockholm, Sweden. ellinor.hammarstrom@telia.com; 2010;89(10):1316–25. Available from: http://ovidsp.ovid.com/ovidweb.cgi?T=JS&PAGE=reference&D=medl&NEWS=N&AN=20846064

74. Östensson E, Hellström A-C, Hellman K, Gustavsson I, Gyllensten U, Wilander E, et al. Projected cost-effectiveness of repeat high-risk human papillomavirus testing using self-collected vaginal samples in the Swedish cervical cancer screening program. Acta Obstet Gynecol Scand [Internet]. 2013 Jul [cited 2013 Sep 17];92(7):830–40. Available from: http://onlinelibrary.wiley.com/doi/10.1111/aogs.12143/full

75. Parkin DM. A computer simulation model for the practical planning of cervical cancer screening programmes [Internet]. British journal of cancer. International Agency for Research on Cancer, Lyon France; 1985. p. 551–68. Available from: http://ovidsp.ovid.com/ovidweb.cgi?T=JS&PAGE=reference&D=med2&NEWS=N&AN=3978033

76. Parkin DM, Moss SM. An evaluation of screening policies for cervical cancer in England and Wales using a computer simulation model. [Internet]. Journal of epidemiology and community health. ENGLAND; 1986. p. 143–53. Available from: http://ovidsp.ovid.com/ovidweb.cgi?T=JS&PAGE=reference&D=med2&NEWS=N&AN=3746177

77. Perkins RB, Langrish SM, Stern LJ, Burgess JF, Simon CJ. Impact of patient adherence and test performance on the cost-effectiveness of cervical cancer screening in developing countries: the case of Honduras. [Internet]. Women’s health issues : official publication of the Jacobs Institute of Women's Health. United States: Department of Obstetrics and Gynecology, Boston University Medical Center, 85 E. Concord St. 6th Floor, Boston, MA 02118, USA. rebecca.perkins@bmc.org; 2010. p. 35–42. Available from: http://ovidsp.ovid.com/ovidweb.cgi?T=JS&PAGE=reference&D=medl&NEWS=N&AN=19944623

78. Philips Z, Whynes DKK. Early withdrawal from cervical cancer screening: The question of cost-effectiveness [Internet]. European Journal of Cancer. D.K. Whynes, School of Economics, University of Nottingham, Nottingham NG7 2RD, United Kingdom. E-mail: david.whynes@nottingham.ac.uk: Elsevier Ltd; 2001. p. 1775–80. Available from: http://ovidsp.ovid.com/ovidweb.cgi?T=JS&PAGE=reference&D=emed5&NEWS=N&AN=2001311503

79. Raab SS, Zaleski MS, Silverman JF. The cost-effectiveness of the cytology laboratory and new cytology technologies in cervical cancer prevention. Am J Clin Pathol. 1999;111:259-26:259–66.

80. Raab SS. The cost-effectiveness of cervical-vaginal rescreening. Am J Clin Pathol [Internet]. 1997;108(5):525–36. Available from: http://onlinelibrary.wiley.com/o/cochrane/cleed/articles/NHSEED-21997001451/frame.html

81. Raab SS, Bishop NS, Zaleski MS. Cost effectiveness of rescreening cervicovaginal smears. Am J Clin Pathol [Internet]. UNITED STATES: Department of Pathology and Laboratory Medicine, Allegheny University of Health Sciences, Pittsburgh, PA 15212-4772, USA.; 1999;111:601-60(5):601–9. Available from: http://ovidsp.ovid.com/ovidweb.cgi?T=JS&PAGE=reference&D=med4&NEWS=N&AN=10230350

82. Raab SS, Steiner AL, Hornberger J. The cost-effectiveness of treating women with a cervical vaginal smear diagnosis of atypical squamous cells of undetermined significance. Am J Obstet Gynecol [Internet]. UNITED STATES: Department of Pathology, University of Iowa Hospitals and Clinics, Iowa City, USA.; 1998;179(2):411–20. Available from: http://ovidsp.ovid.com/ovidweb.cgi?T=JS&PAGE=reference&D=med4&NEWS=N&AN=9731847

83. Radensky PW, Mango LJ. Interactive neural network-assisted screening: an economic assessment. Acta Cytol. UNITED STATES: McDermott, Will, & Emery, Miami, Florida, USA.; 1998;42:246–52.

84. Raffle AE, Alden B, Quinn MA, Babb PJ, Brett MT. Outcomes of screening to prevent cancer: Analysis of cumulative incidence of cervical abnormality and modelling of cases and deaths prevented [Internet]. British Medical Journal. A.E. Raffle, Avon Health Authority, King Square House, Bristol BS2 8EE, United Kingdom. E-mail: angela.raffle@bristolnorth-pct.nhs.uk: BMJ Publishing Group; 2003. p. 901–4. Available from: http://ovidsp.ovid.com/ovidweb.cgi?T=JS&PAGE=reference&D=emed6&NEWS=N&AN=2003180104

85. Sato S, Matunaga G, Tsuji I, Yajima A, Sasaki H. Determining the cost-effectiveness of mass screening for cervical cancer using common analytic models (Structured abstract). Acta Cytol [Internet]. 1999;43(6):1006–14. Available from: http://onlinelibrary.wiley.com/o/cochrane/cleed/articles/NHSEED-21999002191/frame.html

86. Sawaya G, McConnell KJ, Kulasingam SL, Lawson HW, Kerlikowske K, Melnikow J, et al. Risk of cervical cancer associated with extending the interval between cervical-cancer screenings. N Engl J Med [Internet]. 2003 [cited 2013 Sep 13];349(16):1501–9. Available from: http://scholar.google.com/scholar?hl=en&btnG=Search&q=intitle:new+england+journal#2

87. Schechter CB. Cost-effectiveness of rescreening conventionally prepared cervical smears by PAPNET testing (Structured abstract). Acta Cytol [Internet]. UNITED STATES: Department of Community Medicine, Mt. Sinai School of Medicine, New York, New York, USA.; 1996;40(6):1272–82. Available from: http://onlinelibrary.wiley.com/o/cochrane/cleed/articles/NHSEED-21997006329/frame.html

88. Sheriff SK, Petry KU, Ikenberg H, Crouse G, Mazonson PD, Santas CC. An Economic Analysis of Human Papillomavirus Triage for the Management of Women with Atypical and Abnormal Pap Smear Results in Germany. European Journal of Health Economics. 2007. p. 153–60.

89. Sherlaw-Johnson C, Philips Z. An evaluation of liquid-based cytology and human papillomavirus testing within the UK cervical cancer screening programme [Internet]. British Journal of Cancer. England: Clinical Operational Research Unit, University College London, Department of Mathematics, University College London, Gower Street, London WC1E 6BT, UK. c.sherlaw-johnson@ucl.ac.uk; 2004. p. 84–91. Available from: http://ovidsp.ovid.com/ovidweb.cgi?T=JS&PAGE=reference&D=med4&NEWS=N&AN=15162150

90. Sherlaw-Johnson C, Gallivan S. The planning of cervical cancer screening programmes in Eastern Europe: is viral testing a suitable alternative to smear testing? (Structured abstract) [Internet]. Health Care Management Science. 2000. p. 323–9. Available from: http://onlinelibrary.wiley.com/o/cochrane/cleed/articles/NHSEED-22001008095/frame.html

91. Sherlaw-Johnson C, Gallivan S, Jenkins D. Evaluating cervical cancer screening programmes for developing countries. Int J Cancer. 1997;72:210-216.

92. Sherlaw-Johnson C, Gallivan S, Jenkins D. Withdrawing low risk women from cervical screening programmes: Mathematical modelling study [Internet]. British Medical Journal. C. Sherlaw-Johnson, Clinical Operational Research Unit, Department of Mathematics, University College London, London WC1E 6BT, United Kingdom. E-mail: c.sherlaw-johnson@ucl.ac.uk: BMJ Publishing Group; 1999. p. 356–61. Available from: http://ovidsp.ovid.com/ovidweb.cgi?T=JS&PAGE=reference&D=medc&NEWS=N&AN=9933195

93. Sherlaw-Johnson C, Gallivan S, Jenkins D, Jones MH. Cytological screening and management of abnormalities in prevention of cervical cancer: an overview with stochastic modelling. [Internet]. Journal of clinical pathology. ENGLAND: Department of Statistical Science, University College London.; 1994. p. 430–5. Available from: http://ovidsp.ovid.com/ovidweb.cgi?T=JS&PAGE=reference&D=med3&NEWS=N&AN=8027396

94. Shi J-FF, Canfell K, Lew J-BB, Zhao F-HH, Legood R, Ning Y, et al. Evaluation of primary HPV-DNA testing in relation to visual inspection methods for cervical cancer screening in rural China: an epidemiologic and cost-effectiveness modelling study (Provisional abstract) [Internet]. BMC Cancer. England: Department of Cancer Epidemiology, Cancer Institute, Chinese Academy of Medical Sciences, Peking Union Medical College, 17, South Panjiayuan LN, PO Box 2258, Beijing 100021, China.; 2011. p. 239. Available from: http://onlinelibrary.wiley.com/o/cochrane/cleed/articles/NHSEED-22011001405/frame.html

95. Shun-Zhang Y, Miller AB, Sherman GJ. Optimising the age, number of tests, and test interval for cervical screening in Canada. [Internet]. Journal of epidemiology and community health. ENGLAND; 1982. p. 1–10. Available from: http://ovidsp.ovid.com/ovidweb.cgi?T=JS&PAGE=reference&D=med2&NEWS=N&AN=7069349

96. Siebert U, Sroczynski G, Hillemanns P, Engel J, Stabenow R, Stegmaier C, et al. The German cervical cancer screening model: development and validation of a decision-analytic model for cervical cancer screening in Germany. Eur J Public Health [Internet]. 2006 Apr [cited 2014 Jul 4];16(2):185–92. Available from: http://www.ncbi.nlm.nih.gov/pubmed/16469759

97. Smith BL, Lee M, Leader S, Wertlake P. Economic impact of automated primary screening for cervical cancer. J Reprod Med [Internet]. UNITED STATES: Research Solutions, Redlands, CA 92373-5802, USA.; 1999;44(6):518–28. Available from: http://ovidsp.ovid.com/ovidweb.cgi?T=JS&PAGE=reference&D=med4&NEWS=N&AN=10394546

98. Sroczynski G, Schnell-Inderst P, Muhlberger N, Lang K, Aidelsburger P, Wasem J, et al. Cost-effectiveness of primary HPV screening for cervical cancer in Germany: a decision analysis (Structured abstract) [Internet]. European Journal of Cancer. England: Department of Public Health and Health Technology Assessment, UMIT - University for Health Sciences, Medical Informatics and Technology, Eduard Wallnoefer Center I, A-6060 Hall iT, Austria.; 2011. p. 1633–46. Available from: http://ovidsp.ovid.com/ovidweb.cgi?T=JS&PAGE=reference&D=medl&NEWS=N&AN=21482103

99. Stout NK, Goldhaber-Fiebert JD, Ortendahl JD, Goldie SJ. Trade-offs in cervical cancer prevention: balancing benefits and risks. [Internet]. Archives of internal medicine. United States: Program in Health Decision Science, Department of Health Policy and Management, Harvard School of Public Health, 718 Huntington Ave, Second Floor, Boston, MA 02115, USA. natasha_stout@hms.harvard.edu; 2008. p. 1881–9. Available from: http://ovidsp.ovid.com/ovidweb.cgi?T=JS&PAGE=reference&D=med4&NEWS=N&AN=18809815

100. Straughn JM, Numnum TM, Rocconi RP, Iii CAL, Partridge EE. A Cost-Effectiveness Analysis of Screening Strategies for Cervical. J Low Genit Tract Dis. 2004;8(4):280–4.

101. Suba EJ, Nguyen CH, Nguyen BD, Raab SS, Project VCCP. De novo establishment and cost-effectiveness of Papanicolaou cytology screening services in the Socialist Republic of Vietnam. [Internet]. Cancer. United States: Kaiser Permanente Medical Center, Redwood City, California, USA. eric.suba@kp.org; 2001. p. 928–39. Available from: http://ovidsp.ovid.com/ovidweb.cgi?T=JS&PAGE=reference&D=med4&NEWS=N&AN=11251944

102. Taylor LA, Sorensen S V, Ray NF, Halpern MT, Harper DM. Cost-effectiveness of the conventional Papanicolaou test with a new adjunct to cytological screening for squamous cell carcinoma of the uterine cervix and its precursors (Structured abstract). Arch Fam Med [Internet]. UNITED STATES: MEDTAP International, Inc, Bethesda, MD 20814, USA.; 2000;9(8):713–21. Available from: http://onlinelibrary.wiley.com/o/cochrane/cleed/articles/NHSEED-22000001385/frame.html

103. Van Ballegooijen M, van den Akker-van Marle E, Patnick J, Lynge E, Arbyn M, Anttila A, et al. Overview of important cervical cancer screening process values in European Union (EU) countries, and tentative predictions of the corresponding effectiveness and cost-effectiveness [Internet]. European Journal of Cancer. ENGLAND: Department of Public Health, Faculty of Medicine and Health Sciences, Erasmus University Rotterdam, PO Box 1738, 3000 DR, Rotterdam, The Netherlands. vanmarle@mgz.fgg.eur.nl; 2000. p. 2177–88. Available from: http://onlinelibrary.wiley.com/o/cochrane/cleed/articles/NHSEED-22001000014/frame.html

104. Van Ballegooijen M, Habbema JDFD, van Oortmarssen GJJ, Koopmanschap MAA, Lubbe JTNT, van Agt HM. M. Preventive pap-smears:balancing costs, risks and benefits. Br J Cancer [Internet]. ENGLAND: Department of Public Health and Social Medicine, Erasmus University Rotterdam, The Netherlands.; 1992;65:930-933(6):930–3. Available from: http://ovidsp.ovid.com/ovidweb.cgi?T=JS&PAGE=reference&D=med3&NEWS=N&AN=1616867

105. Van Ballegooijen M, Van den Akker-van Marle MEE, Warmerdam PGG, Meijer CJLMJ, Walboomers JMMM, Habbema JDFD. Present evidence on the value of HPV testing for cervical cancer screening: a model-based exploration of the (cost-)effectiveness. Br J Cancer [Internet]. SCOTLAND: Department of Public Health, Erasmus University, Rotterdam, The Netherlands.; 1997;76(5):651-(5):651–7. Available from: http://ovidsp.ovid.com/ovidweb.cgi?T=JS&PAGE=reference&D=med4&NEWS=N&AN=9303366

106. Van den Akker-van Marle ME, van Ballegooijen M, van Oortmarssen GJ, Boer R, Habbema JDF. Cost-effectiveness of cervical cancer screening: comparison of screening policies. [Internet]. Journal of the National Cancer Institute. United States: Department of Public Health, Faculty of Medicine and Health Sciences, Erasmus University Rotterdam, The Netherlands. vanmarle@mgz.fgg.eur.nl; 2002. p. 193–204. Available from: http://ovidsp.ovid.com/ovidweb.cgi?T=JS&PAGE=reference&D=med4&NEWS=N&AN=11830609

107. Van Oortmarssen GJJ, Habbema JD, van Ballegooijen M. Predicting mortality from cervical cancer after negative smear test results. [Internet]. BMJ (Clinical research ed.). ENGLAND: Department of Public Health and Social Medicine, Medical Faculty, Erasmus University, Rotterdam, Netherlands.; 1992. p. 449–51. Available from: http://ovidsp.ovid.com/ovidweb.cgi?T=JS&PAGE=reference&D=med3&NEWS=N&AN=1392957

108. Van Rosmalen J, de Kok IMCM, van Ballegooijen M. Cost-effectiveness of cervical cancer screening: cytology versus human papillomavirus DNA testing (Structured abstract) [Internet]. BJOG. An International Journal of Obstetrics and Gynaecology. England: Department of Public Health, Erasmus MC, University Medical Center, Rotterdam, the Netherlands. j.vanrosmalen@erasmusmc.nl; 2012. p. 699–709. Available from: http://ovidsp.ovid.com/ovidweb.cgi?T=JS&PAGE=reference&D=medc&NEWS=N&AN=22251259

109. Vanni T, Luz PM, Grinsztejn B, Veloso VG, Foss A, Mesa-Frias M, et al. Cervical cancer screening among HIV-infected women: An economic evaluation in a middle-income country [Internet]. International Journal of Cancer. United States: Department of Health Services Research and Policy, Faculty of Public Health and Policy, London School of Hygiene and Tropical Medicine, London, United Kingdom. tazio.vanni@lshtm.ac.uk; 2012. p. E96–104. Available from: http://ovidsp.ovid.com/ovidweb.cgi?T=JS&PAGE=reference&D=medl&NEWS=N&AN=21964797

110. Vanni T, Legood R, Franco EL, Villa LL, Luz PM, Schwartsmann G. Economic evaluation of strategies for managing women with equivocal cytological results in Brazil. [Internet]. International journal of cancer. Journal international du cancer. United States: Department of Health Service Research and Policy, Faculty of Public Health and Policy, London School of Hygiene and Tropical Medicine, London, United Kingdom. tazio.vanni@lshtm.ac.uk; 2011. p. 671–9. Available from: http://ovidsp.ovid.com/ovidweb.cgi?T=JS&PAGE=reference&D=medl&NEWS=N&AN=20886598

111. Vijayaraghavan A, Efrusy MB, Lindeque G, Dreyer G, Santas CC. Cost effectiveness of high-risk HPV DNA testing for cervical cancer screening in South Africa. Gynecol Oncol [Internet]. United States: McKesson Corporation, 15 Hillcrest Avenue, Larkspur, CA 94939, USA. vijayaraghavan_arthi@yahoo.com; 2009;112(2):377(2):377–83. Available from: http://ovidsp.ovid.com/ovidweb.cgi?T=JS&PAGE=reference&D=medl&NEWS=N&AN=19081611

112. Vijayaraghavan A, Efrusy MB, Mayrand M-H, Santas CC, Goggin P. Cost-effectiveness of high-risk human papillomavirus testing for cervical cancer screening in Quebec, Canada [Internet]. Canadian journal of public health. Revue canadienne de sante publique. A. Vijayaraghavan, McKesson Corp., San Francisco, CA, USA.: McKesson Corp., San Francisco, CA, USA. vijayaraghavan_arthi@yahoo.com; 2010. p. 220–5. Available from: http://ovidsp.ovid.com/ovidweb.cgi?T=JS&PAGE=reference&D=medl&NEWS=N&AN=20737813

113. Vijayaraghavan A, Efrusy MB, Goodman KA, Santas CC, Huh WK. Cost-effectiveness of using human papillomavirus 16/18 genotype triage in cervical cancer screening. [Internet]. Gynecologic oncology. United States: McKesson Corp., San Francisco, California, USA. vijayaraghavan_arthi@yahoo.com; 2010. p. 237–42. Available from: http://ovidsp.ovid.com/ovidweb.cgi?T=JS&PAGE=reference&D=medl&NEWS=N&AN=20713299

114. Voko Z, Nagyjanosi L, Margitai B, Kovi R, Toth Z, Laszlo D, et al. Modeling cost-effectiveness of cervical cancer screening in hungary [Internet]. Value in Health. Z. Vok, Department of Health Policy and Health Economics, Institute of Economics, Etvs Lornd University, Pzmny Pter stny 1/a, H-1117 Budapest, Hungary. E-mail: voko@caesar.elte.hu: Elsevier Ltd (Langford Lane, Kidlington, Oxford OX5 1GB, United Kingdom); 2012. p. 39–45. Available from: http://ovidsp.ovid.com/ovidweb.cgi?T=JS&PAGE=reference&D=medl&NEWS=N&AN=22264970

115. Willis BH, Barton P, Pearmain P, Bryan S, Hyde C. Cervical screening programmes: can automation help? Evidence from systematic reviews, an economic analysis and a simulation modelling exercise applied to the UK (Structured abstract) [Internet]. Health technology assessment (Winchester, England). England: ARIF, Department of Public Health and Epidemiology, University of Birmingham, UK.; 2005. p. 1–iii. Available from: http://onlinelibrary.wiley.com/o/cochrane/clhta/articles/HTA-32005000187/frame.html

116. Woo PPS, Kim JJ, Leung GM. What is the most cost-effective population-based cancer screening program for Chinese women? [Internet]. Journal of Clinical Oncology. United States: American Society of Clinical Oncology; 2007. p. 617–24. Available from: http://ovidsp.ovid.com/ovidweb.cgi?T=JS&PAGE=reference&D=med4&NEWS=N&AN=17308266

117. Woo PPS, Thach TQ, Choy STB, McGhee SM, Leung GM. Modelling the impact of population-based cytologic screening on cervical cancer incidence and mortality in Hong Kong: an age--period--cohort approach. [Internet]. British journal of cancer. England: Department of Community Medicine and School of Public Health, University of Hong Kong, 21 Sassoon Road, Pokfulam, Hong Kong, China.; 2005. p. 1077–83. Available from: http://ovidsp.ovid.com/ovidweb.cgi?T=JS&PAGE=reference&D=med4&NEWS=N&AN=16205695

118. Wu J. Cervical cancer prevention through cytologic and human papillomavirus DNA screening in Hong Kong Chinese women. [Internet]. Hong Kong medical journal = Xianggang yi xue za zhi / Hong Kong Academy of Medicine. China: School of Public Health, The University of Hong Kong. joewu@hku.hk; 2011. p. 20–4. Available from: http://ovidsp.ovid.com/ovidweb.cgi?T=JS&PAGE=reference&D=medl&NEWS=N&AN=21673355

1. Econ, economic analysis; Epid, epidemiological analysis; Cyt, cytology; HPV, HPV DNA testing; SS, HPV DNA self-sampling; GT, HPV DNA genotyping; Auto, automated reading [↑](#footnote-ref-1)
