# Supplementary material for: Systematic review of model-based cervical screening evaluations
Source: BMC Cancer. 2015 May 1;15:334. doi: 10.1186/s12885-015-1332-8 (PMC4419493; doi:10.1186/s12885-015-1332-8)
Supplement: Additional file 5: — Main characteristics of studies focused on screening interventions alongside vaccination. Table with main data extracted for included studies on screening and vaccination interventions. [file 12885_2015_1332_MOESM5_ESM.docx]

**Additional material 5. Main characteristics of studies focused on screening interventions alongside vaccination (n=35)**

| First Author | Year | Country | Econ/Epid | Aim | Technologies | Main findings | Type of model | Calibration |
| --- | --- | --- | --- | --- | --- | --- | --- | --- |
| Accetta[1] | 2010 | Italy | Econ | TechIntro | Cyt vs HPV | HPV/Cyt5y +/-Vac+ | Static, Deterministic, Aggregate | Formal method |
| Campos[2] | 2012 | Kenya, Eastern Africa, Mozambique, Tanzania, Uganda, Zimbabwe | Econ | TechIntro | HPV vs VIA | HPV3x+, Vac&HPV+ | Static, Stochastic, Individual | Formal method |
| Canfell[3] | 2011 | China | Econ | ScreenIntro | HPV vs HPV | Vac&CareHPV2x(30-59)+ | Dynamic, Deterministic, Aggregate | Visual inspection |
| Chen[4] | 2011 | Taiwan | Econ | TechIntro | Cyt vs HPV | Pap+; HPV&Cyt+ | Static, Stochastic, Individual | Not reported |
| Coupe[5] | 2012 | Netherlands | Econ | TechIntro | Cyt vs HPV | Vac&HPV/Cyt+ | Static, Stochastic, Individual | Visual inspection |
| Coupe[6] | 2009 | Netherlands | Econ | TechIntro | Cyt vs HPV | Vac&HPV/Cyt=Vac&Cyt/HPV>30 | Static, Stochastic, Individual | Unspecified method |
| Coupe[7] | 2009 | Netherlands | Econ | Algorithm | Cyt vs Cyt | Vac&Cyt+ | Static, Stochastic, Individual | Formal method |
| Crowcroft[8] | 2012 | Canada | Epid | Algorithm | Cyt vs Cyt | Vac&Cyt+ | Static, Deterministic, Aggregate | Not reported |
| de Blasio[9] | 2012 | Norway | Epid | Algorithm | Cyt vs Cyt | Vac&Screen+; 30-;59-; 5y-; (25-69)3y+ | Dynamic, Deterministic, Aggregate | Visual inspection |
| Diaz[10] | 2008 | India | Econ | ScreenIntro | Cyt vs HPV vs VIA | Vac&2visitHPV3x(35,40,45)+ | Static, Stochastic, Individual | Formal method |
| Diaz[11] | 2010 | Spain | Econ | ScreenIntro | Cyt vs HPV | Screen+; Vac&HPV/Cyt>30+ | Static, Stochastic, Individual | Formal method |
| Ezat[12] | 2010 | Malaysia | Econ | Algorithm | Cyt vs Cyt | Screen&QVvac+ | Static, Deterministic, Aggregate | Not reported |
| Ginsberg[13] | 2012 | Sub-Saharan Africa, South East Asia | Econ | ScreenIntro | Cyt vs HPV vs VIA | Vac-, Cyt or VIA+ | Static, Deterministic, Aggregate | Not reported |
| Ginsberg[14] | 2009 | EMRO, SEARO, AMRO, WPRO, AFRO, Eastern Europe | Econ | ScreenIntro | Cyt vs HPV vs VIA | HIC:Vac&anyScreen+; LMIC:Vac&Pap3or5y+ | Static, Deterministic, Aggregate | Not reported |
| Ginsberg[15] | 2007 | Israel | Econ | ScreenIntro | Cyt vs HPV vs VIA | HPV-, VIA-, Vac&Pap5y+ | Static, Deterministic, Aggregate | Not reported |
| Goldhaber-Fiebert[16] | 2008 | USA | Econ | TechIntro | Cyt vs HPV | HPV/Cyt(>30)+Cyt/HVP(<30)+/-Vac+ | Static, Stochastic, Individual | Formal method |
| Goldhaber-Fiebert[17] | 2007 | USA | Epid | Algorithm | Cyt vs Cyt | Vac&Screen+; (18-70)5y+ | Static, Stochastic, Individual | Formal method |
| Goldie[18] | 2004 | USA | Econ | TechIntro | Cyt vs HPV | Vac&ConvC(>25)3y+ | Static, Deterministic, Aggregate | Unspecified method |
| Goldie[19] | 2011 | USA | Econ | TechIntro | Cyt vs HPV | Vac&HPV/Cyt>30+ | Static, Stochastic, Individual | Unspecified method |
| Goldie[20] | 2008 | Latin America, Caribbean (Argentina, Chile, Colombia, Mexico, Peru) | Econ | ScreenIntro | Cyt vs HPV | Vac&2-visit HPV3x(>30)+ | Static, Stochastic, Individual | Formal method |
| Goldie[21] | 2007 | Brazil | Econ | ScreenIntro | Cyt vs HPV | Vac&Cyt+ | Static, Stochastic, Individual | Formal method |
| Goldie[22] | 2008 | Developing countries | Econ | ScreenIntro | Cyt vs HPV | Vac&HPV+ | Static, Stochastic, Individual | Not reported |
| Gutierrez-Delgado[23] | 2008 | Mexico | Econ | TechIntro | Cyt vs HPV | Vac&HPV&Cyt+ | Static, Deterministic, Aggregate | Not reported |
| Kim[24] | 2008 | Vietnam | Econ | ScreenIntro | Cyt vs HPV | Vac&HPV5y+ | Static, Stochastic, Individual | Visual inspection |
| Kim[25] | 2009 | USA | Econ | TechIntro | Cyt vs HPV | Vac>30-; HPV&Cyt-; HPV- | Static, Stochastic, Individual | Formal method |
| Kulasingam[26] | 2003 | USA | Econ | Algorithm | Cyt vs Cyt | Vac&Screen(>24)2y+;3-5y-;18- | Static, Deterministic, Aggregate | Not reported |
| McLay[27] | 2010 | USA, Western Europe | Epid | Algorithm | Cyt vs Cyt | Vac&Screen+; >3y+ | Static, Deterministic, Aggregate | Visual inspection |
| Praditsitthikorn[28] | 2011 | Thailand | Econ | ScreenIntro | Cyt vs VIA | VIA(30-45)Pap(50-60)5y+ | Static, Deterministic, Aggregate | Not reported |
| Reynales-Shigematsu[29] | 2009 | Mexico | Econ | Algorithm | Cyt vs Cyt | Vac+, Vac&Cyt- | Static, Deterministic, Aggregate | Visual inspection |
| Rogoza[30] | 2008 | Canada, Netherlands, Taiwan, UK, USA | Econ | TechIntro | Cyt vs HPV | Vac&Cyt/HPV-;Vac&Cyt+ | Static, Deterministic, Aggregate | Unspecified method |
| Sharma[31] | 2012 | Thailand | Econ | ScreenIntro | Cyt vs HPV vs VIA | Vac&HPV5x(>35)+ | Static, Stochastic, Individual | Formal method |
| Sopina[32] | 2011 | New Zealand | Econ | Algorithm | Cyt vs Cyt | Vac&Screen(30-60)5y+; 20-; 69-; 3y- | Static, Deterministic, Aggregate | Not reported |
| Tully[33] | 2012 | Canada | Econ | Algorithm | Cyt vs Cyt | CatchUp+; 25+; 18-;21- | Dynamic, Deterministic, Aggregate | Visual inspection |
| Wong[34] | 2009 | Australia | Econ | Algorithm | Cyt vs Cyt | Vac-;LBC-;ConvCyt 1y+ | Static, Deterministic, Aggregate | Not reported |
| Yamamoto[35] | 2012 | Japan | Econ | Algorithm | Cyt vs Cyt | Vac&Cyt+ | Static, Deterministic, Aggregate | Unspecified method |

# References

1. Accetta G, Biggeri A, Carreras G, Lippi G, Carozzi FM, Confortini M, et al. Is human papillomavirus screening preferable to current policies in vaccinated and unvaccinated women? A cost-effectiveness analysis [Internet]. Journal of Medical Screening England: Biostatistics Unit, ISPO Cancer Research and Prevention Institute, Via Cosimo il Vecchio 2, 50139 Florence, Italy. g.accetta@ispo.toscana.it; 2010 p. 181–9. Available from: http://ovidsp.ovid.com/ovidweb.cgi?T=JS&PAGE=reference&D=medl&NEWS=N&AN=21258128

2. Campos NG, Kim JJ, Castle PE, Ortendahl JD, O’Shea M, Diaz M, et al. Health and economic impact of HPV 16/18 vaccination and cervical cancer screening in Eastern Africa. [Internet]. International journal of cancer. Journal international du cancer. United States: Center for Health Decision Science, Department of Health Policy and Management, Harvard School of Public Health, Boston, MA, USA. gastin@post.harvard.edu; 2012. p. 2672–84. Available from: http://ovidsp.ovid.com/ovidweb.cgi?T=JS&PAGE=reference&D=medl&NEWS=N&AN=21717458

3. Canfell K, Shi J-FF, Lew J-BB, Walker R, Zhao F-HH, Simonella L, et al. Prevention of cervical cancer in rural China: evaluation of HPV vaccination and primary HPV screening strategies [Internet]. Vaccine. Netherlands: Cancer Epidemiology Research Unit, Cancer Council NSW, Woolloomooloo, New South Wales, Australia. karenc@nswcc.org.au; 2011. p. 2487–94. Available from: http://ovidsp.ovid.com/ovidweb.cgi?T=JS&PAGE=reference&D=medl&NEWS=N&AN=21211586

4. Chen M-KK, Hung H-FF, Duffy S, Yen AM-F, Chen H-HH. Cost-effectiveness analysis for Pap smear screening and human papillomavirus DNA testing and vaccination [Internet]. Journal of Evaluation in Clinical Practice. England: Institute of Preventive Medicine, College of Public Health, National Taiwan University, Taipei, Taiwan.; 2011. p. 1050–8. Available from: http://onlinelibrary.wiley.com/o/cochrane/cleed/articles/NHSEED-22012010126/frame.html

5. Coupe VMH, Bogaards JA, Meijer CJLM, Berkhof J. Impact of vaccine protection against multiple HPV types on the cost-effectiveness of cervical screening. Vaccine [Internet]. Netherlands: Department of Epidemiology and Biostatistics, VU University Medical Centre, PO Box 7057, 1007 MB Amsterdam, The Netherlands. v.coupe@vumc.nl; 2012;30(10):181(10):1813–22. Available from: http://ovidsp.ovid.com/ovidweb.cgi?T=JS&PAGE=reference&D=medl&NEWS=N&AN=22240341

6. Coupe VMH, de Melker HE, Snijders PJF, Meijer CJLM, Berkhof J. How to screen for cervical cancer after HPV16/18 vaccination in The Netherlands. Vaccine [Internet]. Netherlands: Department of Clinical Epidemiology and Biostatistics, VU University Medical Centre, Amsterdam, The Netherlands. v.coupe@vumc.nl; 2009;27(37):5111–9. Available from: http://onlinelibrary.wiley.com/o/cochrane/cleed/articles/NHSEED-22009103278/frame.html

7. Coupe VMH, van Ginkel J, de Melker HE, Snijders PJF, Meijer CJLM, Berkhof J. HPV16/18 vaccination to prevent cervical cancer in The Netherlands: model-based cost-effectiveness. Int J Cancer [Internet]. United States: Department of Epidemiology and Biostatistics, VU University Medical Centre, Amsterdam, The Netherlands. v.coupe@vumc.nl; 2009;124(4):970–8. Available from: http://ovidsp.ovid.com/ovidweb.cgi?T=JS&PAGE=reference&D=medl&NEWS=N&AN=19035448

8. Crowcroft NS, Hamid JS, Deeks SL, Frank J. Human papilloma virus vaccination programs reduce health inequity in most scenarios: a simulation study. [Internet]. BMC public health. England: Infectious Diseases, Public Health Ontario, 480 University Avenue, Suite 300, Toronto, Ontario M5G 1V2, Canada. Natasha.crowcroft@oahpp.ca; 2012. p. 935. Available from: http://ovidsp.ovid.com/ovidweb.cgi?T=JS&PAGE=reference&D=medl&NEWS=N&AN=23113881

9. De Blasio BF, Neilson AR, Klemp M, Skjeldestad FE. Modeling the impact of screening policy and screening compliance on incidence and mortality of cervical cancer in the post-HPV vaccination era. [Internet]. Journal of public health (Oxford, England). England: Department of Biostatistics, Institute of Basic Medical Sciences, University of Oslo, PO Box 1122, Blindern 0317, Oslo, Norway. b.f.d.blasio@medisin.uio.no; 2012. p. 539–47. Available from: http://ovidsp.ovid.com/ovidweb.cgi?T=JS&PAGE=reference&D=medl&NEWS=N&AN=22707556

10. Diaz M, Kim JJ, Albero G, de Sanjose S, Clifford G, Bosch FX, et al. Health and economic impact of HPV 16 and 18 vaccination and cervical cancer screening in India. Br J Cancer [Internet]. England: Unit of Infections and Cancer (UNIC), Cancer Epidemiology Research Programme, Catalan Institute of Oncology (ICO), Av. Gran Via, s/n km. 2.7, 08907 L’Hospitalet de Llobregat, Barcelona, Spain.; 2008;99(2):230–8. Available from: http://ovidsp.ovid.com/ovidweb.cgi?T=JS&PAGE=reference&D=med4&NEWS=N&AN=18612311

11. Diaz M, de Sanjose S, Ortendahl J, O’Shea M, Goldie SJ, Bosch FX, et al. Cost-effectiveness of human papillomavirus vaccination and screening in Spain. [Internet]. European journal of cancer (Oxford, England : 1990). England: Unit of Infections and Cancer, Cancer Epidemiology Research Program, Catalan Institute of Oncology, IDIBELL, Av. Gran Via 199-203, 08907 L’Hospitalet de Llobregat, Barcelona, Spain. mireia@iconcologia.net; 2010. p. 2973–85. Available from: http://ovidsp.ovid.com/ovidweb.cgi?T=JS&PAGE=reference&D=medl&NEWS=N&AN=20638840

12. Ezat SWP, Aljunid S. Comparative cost-effectiveness of HPV vaccines in the prevention of cervical cancer in Malaysia. [Internet]. Asian Pacific journal of cancer prevention : APJCP. Thailand: Department of Community Health, UKM Medical Centre, United Nations University-International Institute for Global Health, Kuala Lumpur, Malaysia. sh_ezat@yahoo.com; 2010. p. 943–51. Available from: http://ovidsp.ovid.com/ovidweb.cgi?T=JS&PAGE=reference&D=medl&NEWS=N&AN=21133606

13. Ginsberg GM, Lauer JA, Zelle S, Baeten S, Baltussen R. Cost effectiveness of strategies to combat breast, cervical, and colorectal cancer in sub-Saharan Africa and South East Asia: mathematical modelling study [Internet]. BMJ (Clinical research ed.). England: Department of Medical Technology Assessment, Ministry of Health, Ben Tbai 2, San Simone, Jerusalem, Israel. gary.ginsberg@moh.health.gov.il; 2012. p. e614. Available from: http://ovidsp.ovid.com/ovidweb.cgi?T=JS&PAGE=reference&D=emed10&NEWS=N&AN=22389347

14. Ginsberg GM, Edejer TT-T, Lauer JA, Sepulveda C. Screening, prevention and treatment of cervical cancer: a global and regional generalized cost-effectiveness analysis (Provisional abstract) [Internet]. Vaccine. Netherlands: World Health Organization, Health Systems Financing Department, Costs, Effectiveness, Expenditure and Priority Setting Unit, Geneva, Switzerland. GARY.GINSBERG@moh.health.gov.il; 2009. p. 6060–79. Available from: http://ovidsp.ovid.com/ovidweb.cgi?T=JS&PAGE=reference&D=medl&NEWS=N&AN=19647813

15. Ginsberg GM, Fisher M, Ben-Shahar I, Bornstein J. Cost-utility analysis of vaccination against HPV in Israel. [Internet]. Vaccine. Netherlands: Medical Technology Assessment Sector, Ministry of Health, Ben Tbai 2, San Simone, Jerusalem, Israel. gary.ginsberg@moh.health.gov.il; 2007. p. 6677–91. Available from: http://ovidsp.ovid.com/ovidweb.cgi?T=JS&PAGE=reference&D=med4&NEWS=N&AN=17706844

16. Goldhaber-Fiebert JD, Stout NK, Salomon JA, Kuntz KM, Goldie SJ, Rossi PG, et al. Cost-effectiveness of cervical cancer screening with human papillomavirus DNA testing and HPV-16,18 vaccination. J Natl Cancer Inst [Internet]. United States: Doctoral Program in Health Policy, Decision Science Concentration, Harvard University, Cambridge, MA, USA.; 2008;100(22):1654–5. Available from: http://ovidsp.ovid.com/ovidweb.cgi?T=JS&PAGE=reference&D=med4&NEWS=N&AN=19001608

17. Goldhaber-Fiebert JD, Stout NK, Ortendahl J, Kuntz KM, Goldie SJ, Salomon J a. Modeling human papillomavirus and cervical cancer in the United States for analyses of screening and vaccination. Popul Health Metr [Internet]. 2007 Jan [cited 2013 Nov 13];5:11. Available from: http://www.pubmedcentral.nih.gov/articlerender.fcgi?artid=2213637&tool=pmcentrez&rendertype=abstract

18. Goldie SJ, Kohli M, Grima D, Weinstein MCMC, Wright TC, Bosch FX, et al. Projected clinical benefits and cost-effectiveness of a human papillomavirus 16/18 vaccine. [Internet]. Journal of the National Cancer Institute. United States: Oxford University Press; 2004. p. 604–15. Available from: http://onlinelibrary.wiley.com/o/cochrane/cleed/articles/NHSEED-22004000642/frame.html

19. Goldie SJ, Daniels N. Model-based analyses to compare health and economic outcomes of cancer control: inclusion of disparities. [Internet]. Journal of the National Cancer Institute. United States: Department of Health Policy and Management, Center for Health Decision Science, Harvard School of Public Health, Harvard Global Health Institute, Harvard University, 718 Huntington Ave, 2nd floor, Boston, MA 02115, USA. sue_goldie@harvard.edu; 2011. p. 1373–86. Available from: http://ovidsp.ovid.com/ovidweb.cgi?T=JS&PAGE=reference&D=medc&NEWS=N&AN=21900120

20. Goldie SJ, Diaz M, Constenla D, Alvis N, Andrus JK, Kim S-Y. Mathematical models of cervical cancer prevention in Latin America and the Caribbean. [Internet]. Vaccine. Netherlands: Program in Health Decision Science, Department of Health Policy and Management, Harvard School of Public Health, Boston, MA, USA. sue_goldie@harvard.edu; 2008. p. L59–72. Available from: http://ovidsp.ovid.com/ovidweb.cgi?T=JS&PAGE=reference&D=med4&NEWS=N&AN=18945403

21. Goldie SJ, Kim JJ, Kobus K, Goldhaber-Fiebert JD, Salomon J, O’shea MKH, et al. Cost-effectiveness of HPV 16, 18 vaccination in Brazil. [Internet]. Vaccine. Netherlands: Department of Health Policy and Management, Program in Health Decision Science, Harvard School of Public Health, 718 Huntington Avenue, 2nd Floor, Boston, MA 02115, United States of America. sgoldie@hsph.harvard.edu; 2007. p. 6257–70. Available from: http://ovidsp.ovid.com/ovidweb.cgi?T=JS&PAGE=reference&D=med4&NEWS=N&AN=17606315

22. Goldie SJ, O’Shea M, Diaz M, Kim S-Y. Benefits, cost requirements and cost-effectiveness of the HPV16,18 vaccine for cervical cancer prevention in developing countries: policy implications. [Internet]. Reproductive health matters. Netherlands: Department of Health Policy and Management, Harvard School of Public Health, Boston, MA, USA. phds@hsph.harvard.edu; 2008. p. 86–96. Available from: http://ovidsp.ovid.com/ovidweb.cgi?T=JS&PAGE=reference&D=med4&NEWS=N&AN=19027626

23. Gutierrez-Delgado C, Baez-Mendoza C, Gonzalez-Pier E, de la Rosa AP, Witlen R. [Generalized cost-effectiveness of preventive interventions against cervical cancer in Mexican women: results of a Markov model from the public sector perspective]. [Internet]. Relacion costo-efectividad de las intervenciones preventivas contra el cancer cervical en mujeres mexicanas. Mexico: Unidad de Analisis Economico, Secretaria de Salud, Mexico. cgutierrezd@salud.gob.mx; 2008. p. 107–18. Available from: http://ovidsp.ovid.com/ovidweb.cgi?T=JS&PAGE=reference&D=med4&NEWS=N&AN=18372991

24. Kim JJ, Kobus KE, Diaz M, O’Shea M, Van Minh H, Goldie SJ. Exploring the cost-effectiveness of HPV vaccination in Vietnam: insights for evidence-based cervical cancer prevention policy. [Internet]. Vaccine. Netherlands: Department of Health Policy and Management, Program in Health Decision Science, Harvard School of Public Health, 718 Huntington Avenue, Boston, MA 02115, USA. jkim@hsph.harvard.edu; 2008. p. 4015–24. Available from: http://ovidsp.ovid.com/ovidweb.cgi?T=JS&PAGE=reference&D=med4&NEWS=N&AN=18602731

25. Kim JJ, Ortendahl J, Goldie SJ. Cost-effectiveness of human papillomavirus vaccination and cervical cancer screening in women older than 30 years in the United States. [Internet]. Annals of internal medicine. United States: Department of Health Policy and Management, Center for Health Decision Science, Harvard School of Public Health, 718 Huntington Avenue, Boston, MA 02115, USA.; 2009. p. 538–45. Available from: http://ovidsp.ovid.com/ovidweb.cgi?T=JS&PAGE=reference&D=medl&NEWS=N&AN=19841455

26. Kulasingam SL, Myers ER. Potential health and economic impact of adding a human papillomavirus vaccine to screening programs [Internet]. JAMA : the journal of the American Medical Association. United States: Department of Obstetrics and Gynecology and Center for Clinical Health Policy Research, Duke University, Durham, NC 27705, USA. kulas002@mc.duke.edu; 2003. p. 781–9. Available from: http://ovidsp.ovid.com/ovidweb.cgi?T=JS&PAGE=reference&D=med4&NEWS=N&AN=12915431

27. McLay LA, Foufoulides C, Merrick JRW. Using Simulation-Optimization to Construct Screening Strategies for Cervical Cancer [Internet]. Health Care Management Science. L. A. McLay, Department of Statistical Sciences and Operations Research, Virginia Commonwealth University, P.O. Box 843083, 1015 Floyd Avenue, Richmond, VA 23284, United States. E-mail: lamclay@vcu.edu: Kluwer Academic Publishers; 2010. p. 294–318. Available from: http://ovidsp.ovid.com/ovidweb.cgi?T=JS&PAGE=reference&D=medl&NEWS=N&AN=20963551

28. Praditsitthikorn N, Teerawattananon Y, Tantivess S, Limwattananon S, Riewpaiboon A, Chichareon S, et al. Economic evaluation of policy options for prevention and control of cervical cancer in Thailand (Structured abstract). Pharmacoeconomics [Internet]. New Zealand: Health Intervention and Technology Assessment Program, Ministry of Public Health, Nonthaburi, Thailand. naiyana.p@hitap.net; 2011;29(9):781–806. Available from: http://ovidsp.ovid.com/ovidweb.cgi?T=JS&PAGE=reference&D=medl&NEWS=N&AN=21838332

29. Reynales-Shigematsu LM, Rodrigues ER, Lazcano-Ponce E. Cost-effectiveness analysis of a quadrivalent human papilloma virus vaccine in Mexico. Arch Med Res [Internet]. United States: Instituto Nacional de Salud Publica, Cuernavaca, Morelos, Mexico.; 2009;40(6):503–13. Available from: http://ovidsp.ovid.com/ovidweb.cgi?T=JS&PAGE=reference&D=medl&NEWS=N&AN=19853192

30. Rogoza RM, Ferko N, Bentley J, Meijer CJLM, Berkhof J, Wang K-L, et al. Optimization of primary and secondary cervical cancer prevention strategies in an era of cervical cancer vaccination: a multi-regional health economic analysis. [Internet]. Vaccine. Netherlands: Health Economics and Outcomes Research, i3 Innovus, 1016-A Sutton Drive, Burlington, Ontario, Canada. raina.rogoza@i3innovus.com; 2008. p. F46–58. Available from: http://ovidsp.ovid.com/ovidweb.cgi?T=JS&PAGE=reference&D=med4&NEWS=N&AN=18992382

31. Sharma M, Ortendahl J, van der Ham E, Sy S, Kim JJ. Cost-effectiveness of human papillomavirus vaccination and cervical cancer screening in Thailand. [Internet]. BJOG : an international journal of obstetrics and gynaecology. England: Department of Health Policy and Management, Center for Health Decision Science, Harvard School of Public Health, Boston, MA 02115, USA. msharma04@gmail.com; 2012. p. 166–76. Available from: http://ovidsp.ovid.com/ovidweb.cgi?T=JS&PAGE=reference&D=medl&NEWS=N&AN=21481160

32. Sopina E, Ashton T. Cost-effectiveness of a cervical screening program with human papillomavirus vaccine. [Internet]. International Journal of Technology Assessment in Health Care. England: Health Systems, School of Population Health, Faculty of Medical and Health Sciences, The University of Auckland, Private Bag 92019, Auckland 1142, New Zealand. e.sopina@auckland.ac.nz; 2011. p. 290–7. Available from: http://onlinelibrary.wiley.com/o/cochrane/cleed/articles/NHSEED-22012014540/frame.html

33. Tully SP, Anonychuk AM, Sanchez DM, Galvani AP, Bauch CT. Time for change? An economic evaluation of integrated cervical screening and HPV immunization programs in Canada. [Internet]. Vaccine. Netherlands: Department of Mathematics and Statistics, University of Guelph, Canada.; 2012. p. 425–35. Available from: http://ovidsp.ovid.com/ovidweb.cgi?T=JS&PAGE=reference&D=medl&NEWS=N&AN=22075091

34. Wong G, Howard K, Webster A, Chapman JR, Craig JC. The health and economic impact of cervical cancer screening and human papillomavirus vaccination in kidney transplant recipients. [Internet]. Transplantation. United States: NHMRC Centre for Clinical Research Excellence in Renal Medicine, Children’s Hospital at Westmead, NSW, Australia. germainw@chw.edu.au; 2009. p. 1078–91. Available from: http://ovidsp.ovid.com/ovidweb.cgi?T=JS&PAGE=reference&D=medl&NEWS=N&AN=19352131

35. Yamamoto N, Mori R, Jacklin P, Osuga Y, Kawana K, Shibuya K, et al. Introducing HPV vaccine and scaling up screening procedures to prevent deaths from cervical cancer in Japan: a cost-effectiveness analysis. [Internet]. BJOG : an international journal of obstetrics and gynaecology. England: Department of Global Health Policy, Graduate School of Medicine, University of Tokyo, Japan. nyamamoto@m.u-tokyo.ac.jp; 2012. p. 177–86. Available from: http://ovidsp.ovid.com/ovidweb.cgi?T=JS&PAGE=reference&D=medl&NEWS=N&AN=21794070
