# Supplementary material for: Systematic review of model-based cervical screening evaluations
Source: BMC Cancer. 2015 May 1;15:334. doi: 10.1186/s12885-015-1332-8 (PMC4419493; doi:10.1186/s12885-015-1332-8)
Supplement: Additional file 6: — Number of single- and multiple-country studies per country. Table with number of studies by country and study type. [file 12885_2015_1332_MOESM6_ESM.docx]

**Additional material 6. Number of single- and multiple-country studies per country**

| Country | Single-country studies | Multiple-country studies | Total |
| --- | --- | --- | --- |
| Argentina | - | 1 | 1 |
| Australia | 5 | 1 | 6 |
| Barbados | - | 1 | 1 |
| Belgium | - | 1 | 1 |
| Brazil | 3 | - | 3 |
| Cameroon | 1 | - | 1 |
| Canada | 9 | 3 | 12 |
| Chile | - | 1 | 1 |
| China | 4 | - | 4 |
| Colombia | 1 | 1 | 2 |
| Denmark | 1 | 1 | 2 |
| Germany | 4 | 1 | 5 |
| Greece | 1 | 1 | 2 |
| France | - | 2 | 2 |
| Finland | - | 2 | 2 |
| Honduras | 1 | - | 1 |
| Hong Kong | 3 | - | 3 |
| Hungary | 1 | - | 1 |
| Iceland | - | 1 | 1 |
| India | 1 | 1 | 2 |
| Ireland | - | 1 | 1 |
| Israel | 1 | - | 1 |
| Italy | 1 | 2 | 3 |
| Japan | 3 | 1 | 4 |
| Kenya | - | 2 | 2 |
| Malaysia | 1 | - | 1 |
| Mexico | 3 | 1 | 4 |
| Mozambique | - | 1 | 1 |
| Netherlands | 13 | 3 | 16 |
| New Zealand | 1 | - | 1 |
| Norway | 2 | - | 2 |
| Peru | 1 | 2 | 3 |
| Portugal | 1 | 1 | 2 |
| South Africa | 2 | 1 | 3 |
| Spain | 1 | 1 | 2 |
| Sweden | 3 | 2 | 5 |
| Taiwan | 3 | 1 | 4 |
| Tanzania | - | 1 | 1 |
| Thailand | 3 | 1 | 4 |
| Tunisia | 1 | - | 1 |
| Uganda | - | 1 | 1 |
| UK | 14 | 5 | 19 |
| USA | 44 | 5 | 49 |
| Vietnam | 2 | - | 2 |
| Zimbabwe | - | 1 | 1 |
