# Supplementary material for: Systematic review of model-based cervical screening evaluations
Source: BMC Cancer. 2015 May 1;15:334. doi: 10.1186/s12885-015-1332-8 (PMC4419493; doi:10.1186/s12885-015-1332-8)
Supplement: Additional file 7: — Summary of findings on comparisons of screening technologies. [file 12885_2015_1332_MOESM7_ESM.docx]

**Additional material 7. Summary of findings on comparisons of screening technologies**

| **Intervention/ Comparison** | **No. of studies,**  **Type of analysis** | **Recommended technology (no. of studies, country)** | |
| --- | --- | --- | --- |
| **Cytology** | | | |
| Conventional Cytology vs LBC | 27 (26 Economic, 1 Epidemiological[59]) | | LBC (n=18; Australia[60], China[25], Hong Kong[61], Netherlands[62, 63], Portugal[64], UK[65–67], USA[68–75, 59])  Conventional (n=8, Australia[76][77]†, Canada[29, 78], Netherlands[79], Sweden[80], USA[81, 82]†)  Conventional or LBC (n=1, Canada)[78] |
| Cytology +/- Automation | 7 (6 Economic, 1 Epidemiological[22]) | | Automated cytology (n= 6, USA)[74, 75, 81, 83–85]  Cytology alone (n=1, UK)[22] |
| Cytology +/- Speculoscopy | 1 Economic | | Cytology + Speculoscopy (n=1, USA)[23] |
| **HPV DNA** | | | |
| Cytology vs HPV | 17 Economic | | HPV (n=15; Brazil[86]^a^, Canada[87], Caribbean and Latin America[88]†, Colombia[89], unspecified developing countries[90, 91]†, Eastern Europe[92], European Union[93], Italy[94]†, Netherlands[79]† [95]†, Norway[96], USA[97]†[98]†, Vietnam[99]†)  HPV/cytology triage or Cytology/HPV triage ≥30y (n=1, Netherlands)[100]  Cytology (n=1, Canada)[101] |
| Co-testing vs Cytology vs HPV | 17 (16 Economic, 1 Epidemiological[59]) | | HPV (n= 6; Canada[102], Germany[103, 104], Netherlands[105], Taiwan[106], USA[59])  Co-testing (n=2; USA[107], Mexico[36]†)  Co-testing or HPV (n=3, UK[66], South Africa[108], USA[38]†)  Co-testing or Cytology (n=2, UK, Italy, Netherlands, and France[109], Taiwan[37]†)  Co-testing or HPV or Cytology (n=1, Netherlands)[110]  Cytology (n= 3; Brazil[111]†, Sweden[112], Spain[113]†) |
| Co-testing vs Cytology | 7 Economic | | Co-testing (n=6; Hong Kong[114], UK[67], USA[115–117] [82]^a^  Cytology/HPV triage (n=1, USA)[118] |
| **Triage of cytological abnormalities** |  | |  |
| Repeat Cytology vs HPV vs Co-testing | 1 Economic | | HPV triage (n=1, Netherlands)[62] |
| Repeat Cytology vs HPV vs Immediate treatment | 1 Economic | | HPV triage (n=1,Germany[119]) |
| Repeat Cytology vs HPV vs Immediate colposcopy | 8 Economic | | Colposcopy with biopsy (n=1, Sweden)[120]  HPV triage (n=7; Brazil[121], Canada[78], UK[122], USA[39, 71, 123, 124], Netherlands and Taiwan[39]†)  Cytology triage(n=1, Canada and UK)[39]† |
| **Rapid HPV** | | | |
| Rapid HPV(2x) vs Rapid HPV (1x) | 1 Economic | Rapid HPV (2x) (n=1, China)[26] | |
| Rapid HPV vs VIA | 1 Economic | Rapid HPV (25-49, triennial; 50-64, quinquennial)(n=1, China)[24] | |
| Rapid HPV vs HPV vs Cytology | 1 Economic | Rapid HPV (3x) (n=1, China)[25] | |
| **Self-sampling** | | | |
| Cytology vs HPV vs SS vs VIA | 1 Economic | VIA or HPV; SS vs No screening (n=1, South Africa) [32] | |
| Cytology vs HPV vs Co-testing vs SS | 1 Economic | HPV alone or Co-testing (n=1, Mexico)[125] | |
| Cytology vs HPV vs SS | 1 Economic | SS (n=1, USA)[126] | |
| Cytology vs SS | 1 Economic | SS (≥35y) and cytology (<35y) (n=1, Sweden)[80] | |
| **HPV 16/18 genotyping** | | | |
| Cytology vs HPV vs  Co-testing vs  Co-testing+Genotyping | 1 Economic | Co-testing with HPV 16/18 genotyping triage (n=1, USA)[30] | |
| **VIA** | | | |
| Cytology vs HPV vs SS vs VIA | 1 Economic | VIA or HPV (n=1, South Africa)[32] | |
| Cytology vs HPV vs VIA | 7 Economic | VIA or HPV (n=2, India[18]†, Kenya, Peru, Thailand, and South Africa)[33]  VIA or Cytology (n=1, Sub-Saharan Africa and South East Asia)[127]†  VIA or Co-testing (n=1, Thailand)[128]  VIA in LMIC, any in HIC (n=1, developing countries)[20]†  HPV (n=1, Thailand[31]†)  Cytology (n=1, Israel)[129]† | |
| Cytology vs VIA | 2 Economic | VIA (n=1, Honduras)[34]  VIA (30-45y) and Cytology (50-60y) (n=1, Thailand)[35]† | |
| HPV vs VIA | 1 Economic | HPV (n=1; Kenya, Mozambique, Tanzania, Uganda, Zimbabwe[19]†) | |

^a^HIV-positive women; †screening & vaccination study; Co-testing, combined cytology and HPV DNA testing; HPV, HPV DNA testing; LBC, liquid-based cytology; SS, self-sampling HPV DNA testing; VIA, visual inspection with acetic acid; y, years; 1x, once a lifetime; 2x, twice a lifetime
