# Supplementary material for: Systematic review of model-based cervical screening evaluations
Source: BMC Cancer. 2015 May 1;15:334. doi: 10.1186/s12885-015-1332-8 (PMC4419493; doi:10.1186/s12885-015-1332-8)
Supplement: Additional file 8: — Summary of cervical screening guidelines. [file 12885_2015_1332_MOESM8_ESM.docx]

**Additional material 8. Summary of cervical screening guidelines**

| **Country and Year of publication** | **Recommendation for primary screening** |
| --- | --- |
| Australia 2015[130] | Currently, conventional cytology 2-yearly for women aged ≥18 or 20, or 1-2 years after sexual debut, whichever is later.  In 2016, HPV DNA testing 5-yearly to women aged 25-74 |
| Canada 2013[45] | Cytology^a^ 3-yearly to women aged 25-69 |
| Ireland 2011[131] | LBC 3-yearly to women aged 25-44; 5-yearly to women aged 45-60 |
| Japan 2010[132] | Cytology^a^ recommended 2-yearly to women aged ≥20  Free screening offered 5-yearly to women aged 20–40 |
| Latvia 2009[133] | Cytology^b^ 3-yearly to women aged 25-69 |
| Netherlands 2011[134] | Conventional cytology 5-yearly to women aged 30-60  In 2016, HPV DNA testing 5-yearly to women aged 30-60 |
| New Zealand 2010[135] | Cytology ^a^ 3-yearly to women aged 20-69 |
| Norway 2005[136] | Cytology ^a^ 3-yearly to women aged 25–69  Planning to start introducing HPV DNA primary screening for women aged 34 – 69 in four counties in 2015[137, 138] |
| Portugal 2014[139] | Cytology^a^ 3-yearly to women aged 25-65 or HPV DNA primary testing 5-yearly for women aged 30-65 |
| Singapore 2010[140] | Cytology^a^ 3-yearly to women aged 25–69 |
| South Korea 2012[141] | Cytology^a^ yearly to women aged 20–70 |
| Sweden 2010[142] | LBC 3-yearly to women aged 23-49; 5-yearly to women aged 50-60 |
| Switzerland 2004[143] | Conventional cytology yearly from sexual debut |
| Taiwan 1995[144] | Conventional cytology yearly to women aged ≥30 |
| UK 2012 [145][146] | *England, Northern Ireland and Wales:*  LBC to 25-64; 3-yearly to women aged 25-49; 5-yearly to 50-64  *Scotland:*  LBC 3-yearly to women aged 20-60  In 2016, to change to same schedule as rest of UK |
| USA 2012[147] | Cytology^a^ 3-yearly to women aged 21-65  Optional, Co-testing 5-yearly to women aged 30-65 |

^a^ Conventional or liquid-based; ^b^ Giemsa stain in Leishman modification; Co-testing, combined cytology and HPV DNA testing; LBC, liquid-based cytology
